# Supplementary material for: Inverted U-shaped association between bacillary dysentery and temperature: A new finding using a novel two-stage strategy in multi-region studies
Source: PLoS Negl Trop Dis. 2023 Nov 17;17(11):e0011771. doi: 10.1371/journal.pntd.0011771 (PMC10691710; doi:10.1371/journal.pntd.0011771)
Supplement: S1 Text — Fig B. The pooled average and province-specific ERRs in the overall temperature ranges in sensitivity analysis 1. Fig C. The comparison of ERRs between the classic and novel two-stage strategies in sensitivity analysis 1. Fig D. The curves of pooled average and province-specific BD-temperature ERRs in sensitivity analysis 2. Fig E. The pooled average and province-specific ERRs in the overall temperature ranges in sensitivity analysis 2. Fig F. The comparison of ERRs between the classic and novel two-stage strategies in sensitivity analysis 2. Fig G. The curves of pooled average and province-specific BD-temperature ERRs in sensitivity analysis 3. Fig H. The pooled average and province-specific ERRs in the overall temperature ranges in sensitivity analysis 3. Fig I. The comparison of ERRs between the classic and novel two-stage strategies in sensitivity analysis 3. Fig J. The curves of pooled average and province-specific BD-temperature ERRs in sensitivity analysis 4. Fig K. The pooled average and province-specific ERRs in the overall temperature ranges in sensitivity analysis 4. Fig L. The comparison of ERRs between the classic and novel two-stage strategies in sensitivity analysis 4. Fig M. The curves of pooled average and province-specific BD-temperature ERRs in sensitivity analysis 5. Fig N. The pooled average and province-specific ERRs in the overall temperature ranges in sensitivity analysis 5. Fig O. The pooled average and province-specific ERRs on the original scale within the overall temperature range using the TS-based and B-spline-based strategies. Fig P. Comparison of ERRs on the original scale between the TS-based and B-spline-based strategies in a multi-region study with slightly different exposure ranges (degree of the polynomial was set as 2). Fig Q. Comparison of ERRs on the original scale between the TS-based and B-spline-based strategies in a multi-region study with slightly different exposure ranges (degree of the polynomial was set as 3). Fig R. C [file pntd.0011771.s001.docx]

**Inverted U-shaped association between bacillary dysentery and temperature: a new finding using a novel two-stage strategy in multi-region studies**

## 1. Province-level predictors in meta-regression

Text A. Province-level predictors in meta-regression.

The annual predictors in each province were collected from National Bureau of Statistics via <http://www.stats.gov.cn/>. Then, for each predictor, the mean value across 2004-2017 was calculated in each province. The predictors included rainfall, relative humidity, sunshine duration, per-capital GDP, per-capital wage, urbanization rate, poverty, education, the number of doctors per 10,000 people, the number of health-related worker per 10,000 people, and the number of hospital beds per 10,000 people. Among them, the poverty is measured by the proportion of people whose family incomes are below the lowest living standard and the education is measured by the number of pupils per teacher.

## 2. Sensitivity analyses for the example of BD-temperature ERRS with largely different exposure ranges

### 2.1 Sensitivity analysis 1

Change the knots as 10%, 50%, and 90% quantile temperatures with the latter two being the centered knots.


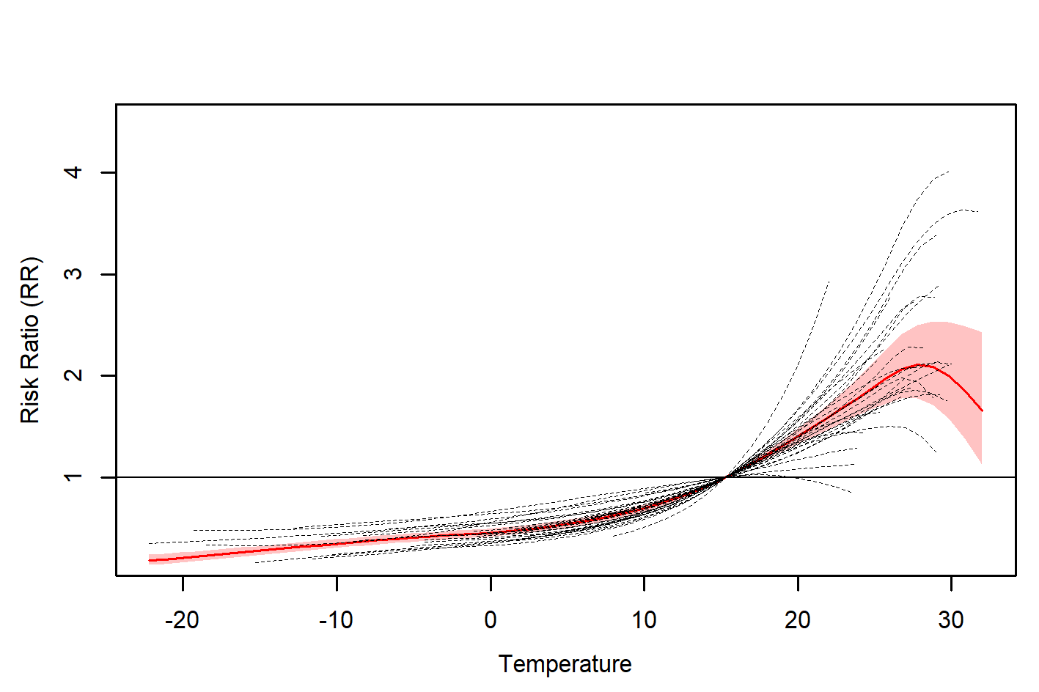


Fig A. The curves of pooled average and province-specific BD-temperature ERRs in sensitivity analysis 1. The red solid line is the average ERR and the shade is 95% confidence interval. The dashed lines are the province-specific ERRs within their corresponding temperature ranges.


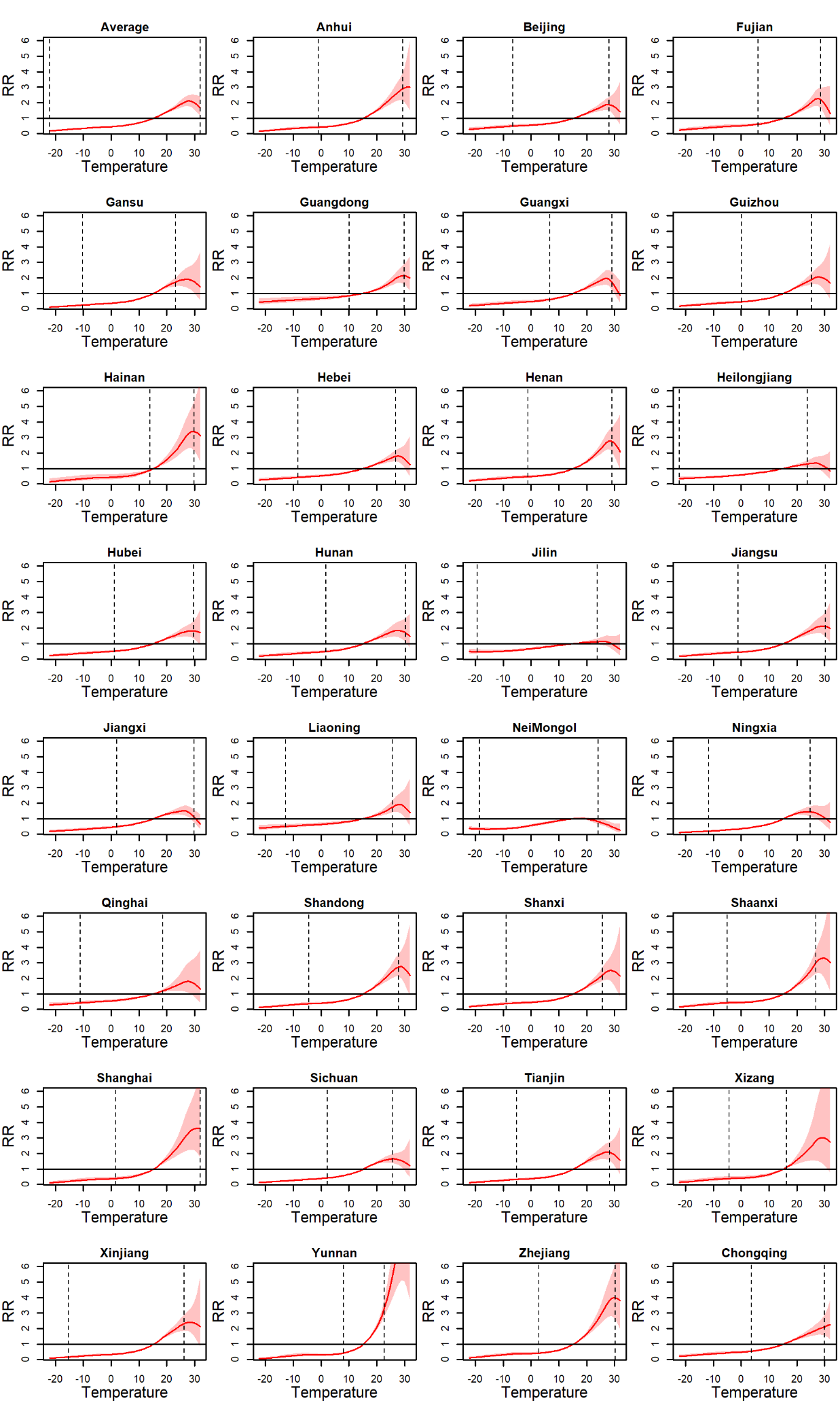


Fig B. The pooled average and province-specific ERRs in the overall temperature ranges in sensitivity analysis 1. The vertical dashed lines indicate the bounds of observed temperature in the corresponding province.


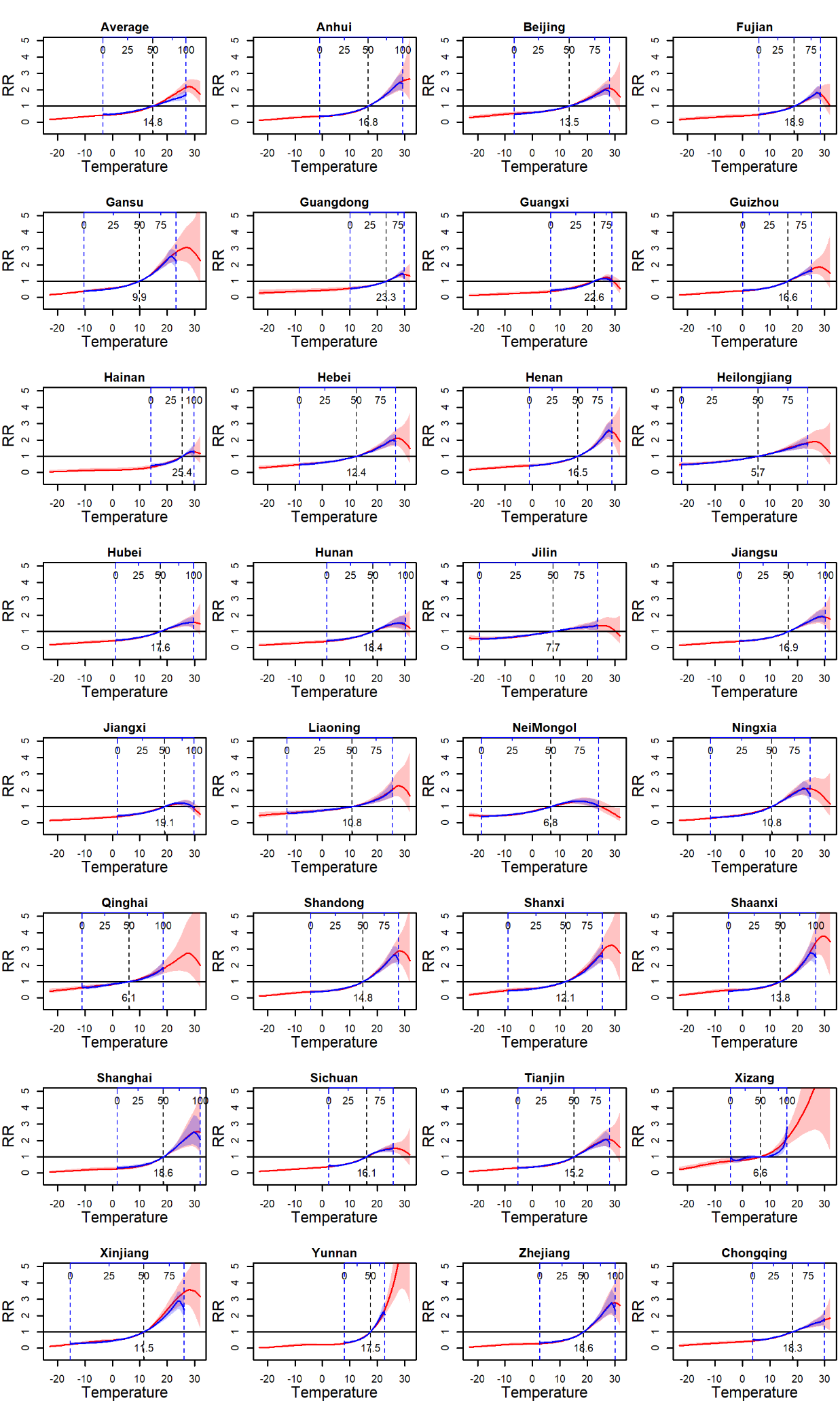


Fig C. The comparison of ERRs between the classic and novel two-stage strategies in sensitivity analysis 1. The blue and red solid curves indicate the ERRs from the classic and novel two-stage strategies, respectively.

### 2.2 Sensitivity analysis 2

Changes degree of freedom of the long-term trend as 4.


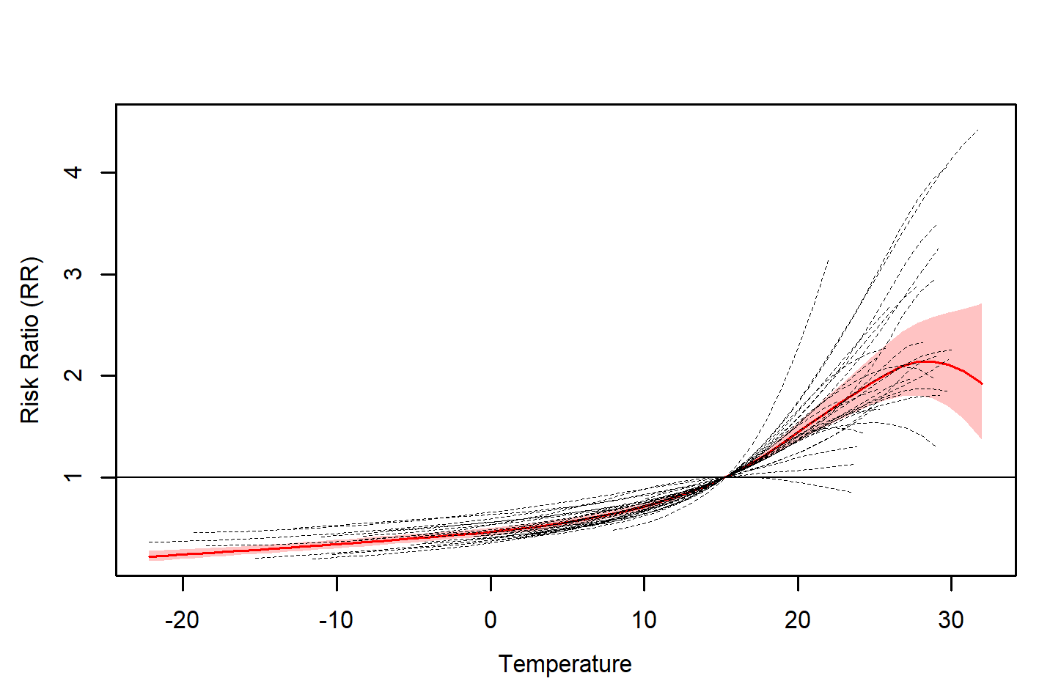


Fig D. The curves of pooled average and province-specific BD-temperature ERRs in sensitivity analysis 2. The red solid line is the average ERR and the shade is 95% confidence interval. The dashed lines are the province-specific ERRs within their corresponding temperature ranges.


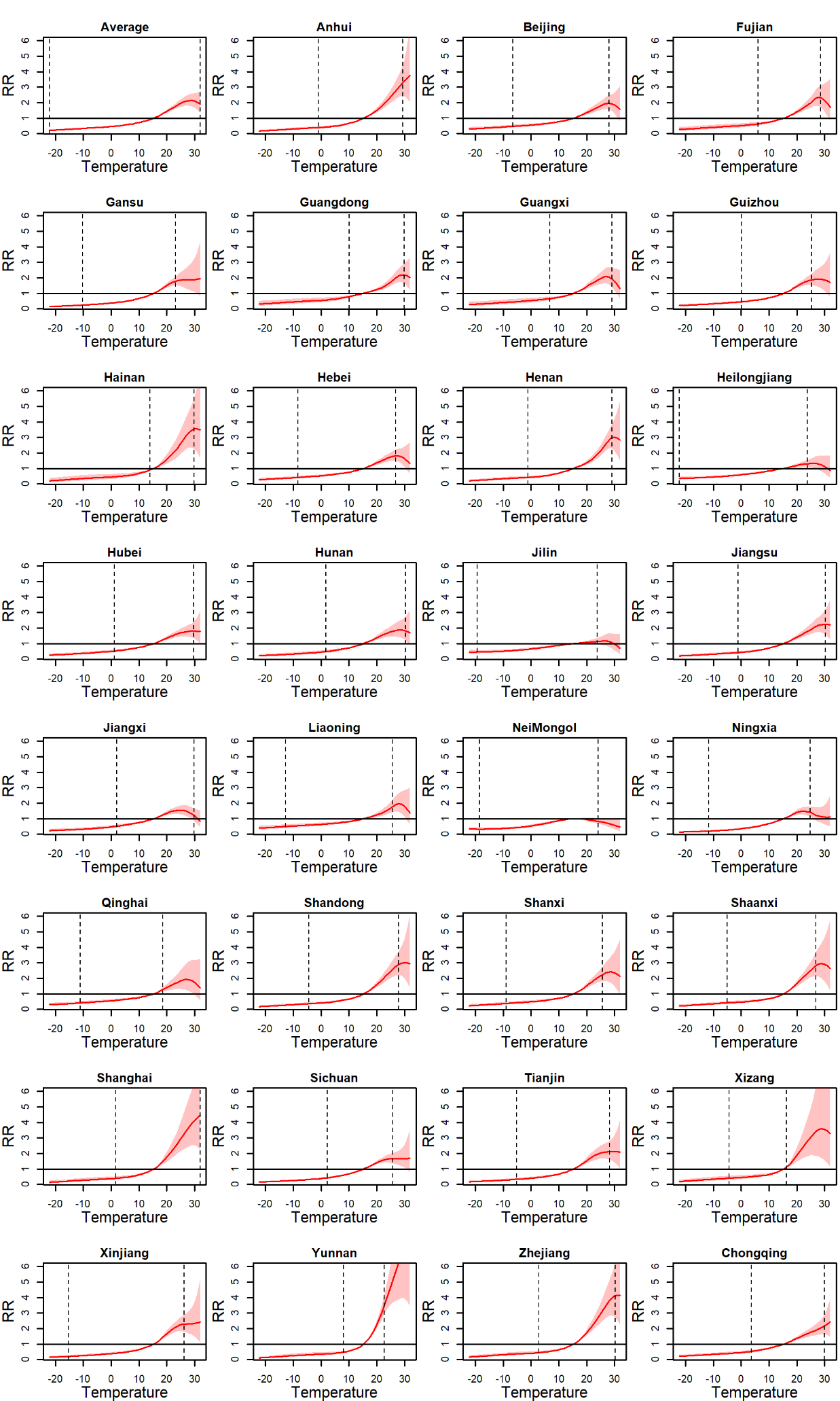


Fig E. The pooled average and province-specific ERRs in the overall temperature ranges in sensitivity analysis 2. The vertical dashed lines indicate the bounds of observed temperature in the corresponding province.


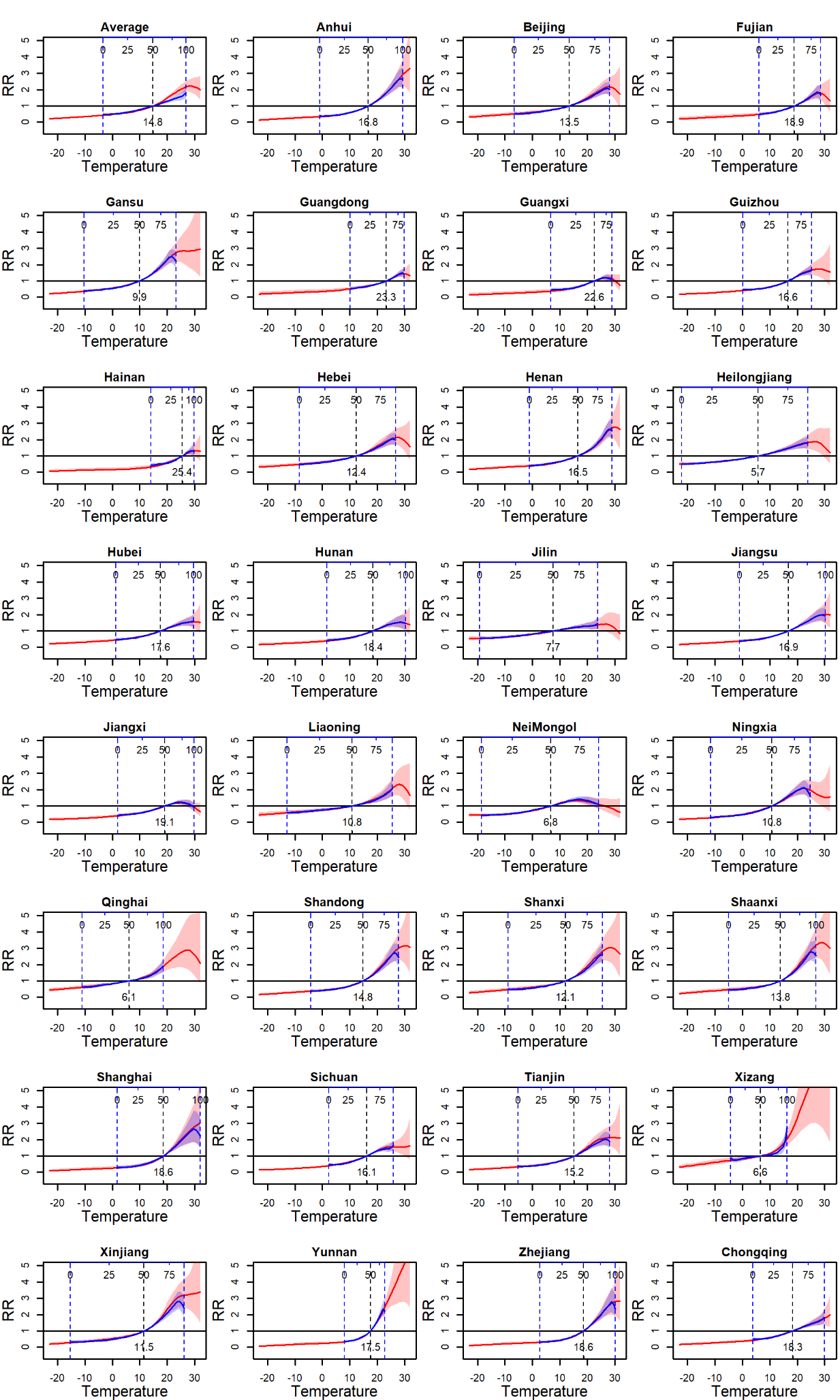


Fig F. The comparison of ERRs between the classic and novel two-stage strategies in sensitivity analysis 2. The blue and red solid curves indicate the ERRs from the classic and novel two-stage strategies, respectively.

### 2.3 Sensitivity analysis 3

Changes degree of freedom of the long-term trends as 8.


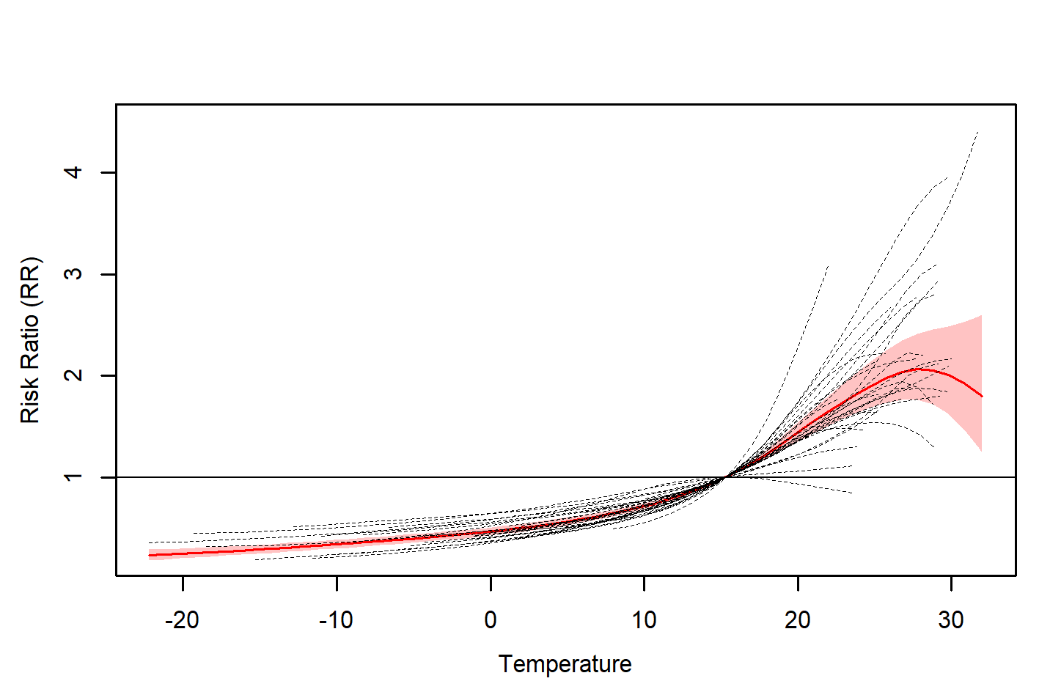


Fig G. The curves of pooled average and province-specific BD-temperature ERRs in sensitivity analysis 3. The red solid line is the average ERR and the shade is 95% confidence interval. The dashed lines are the province-specific ERRs within their corresponding temperature ranges.


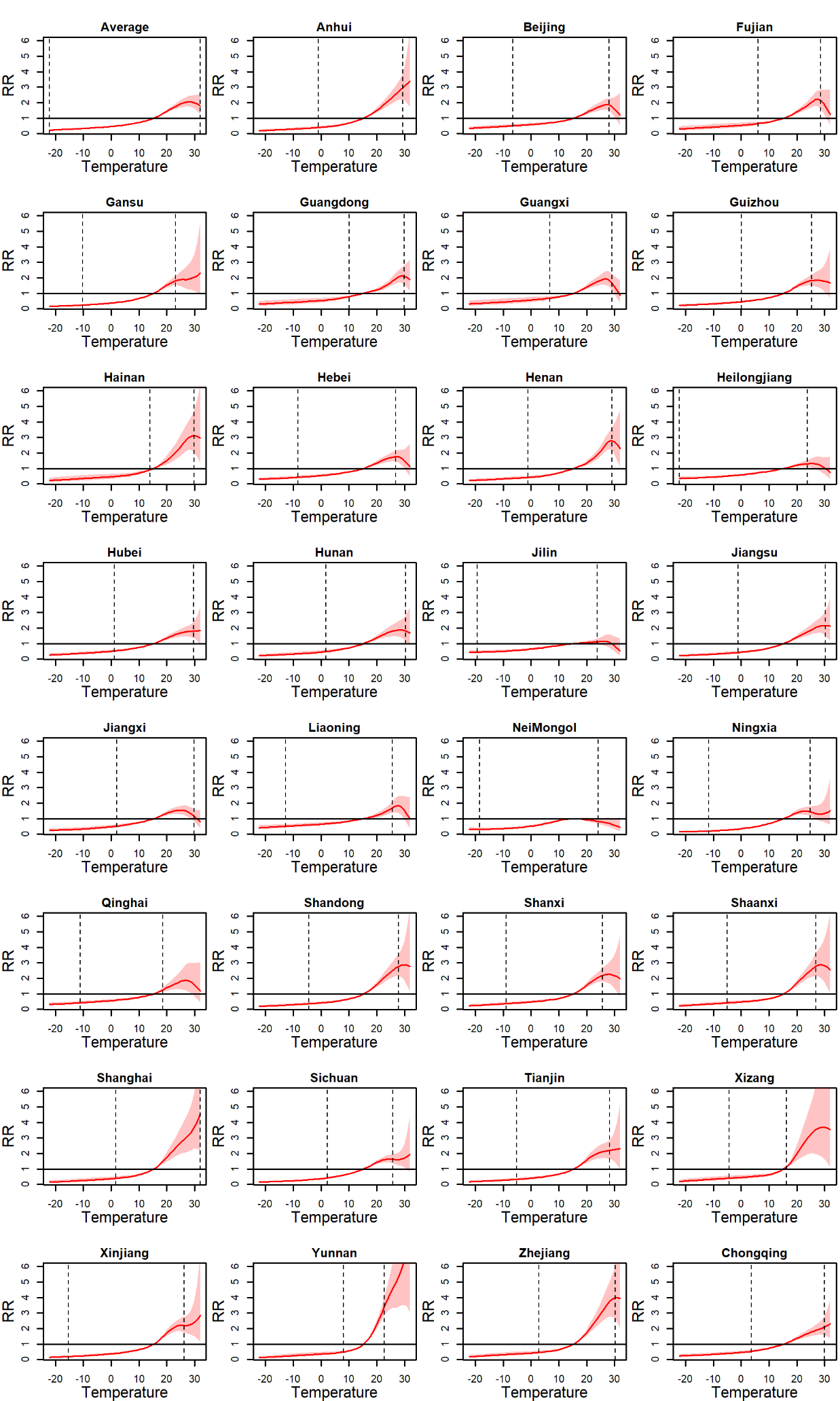


Fig H. The pooled average and province-specific ERRs in the overall temperature ranges in sensitivity analysis 3. The vertical dashed lines indicate the bounds of observed temperature in the corresponding province.


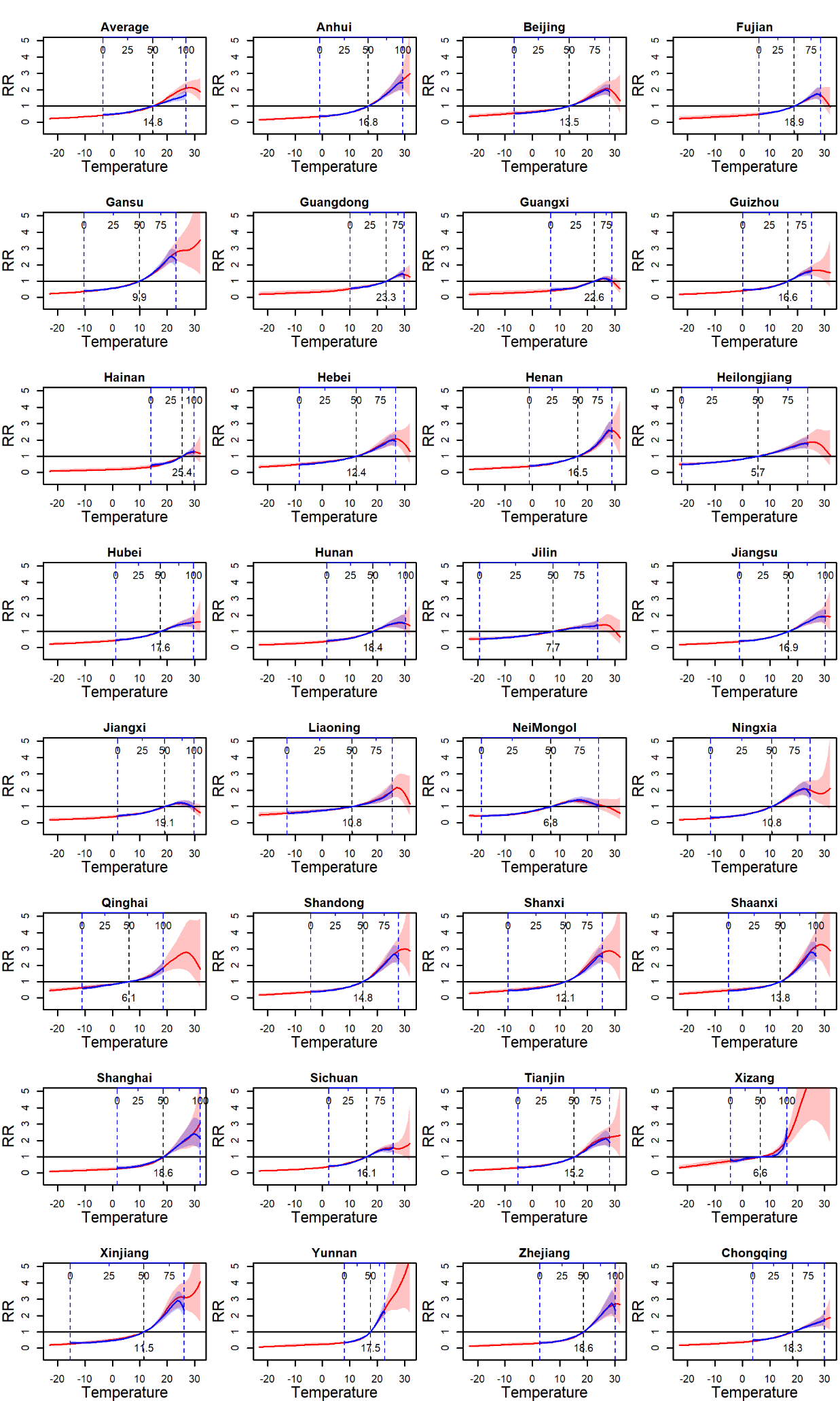


Fig I. The comparison of ERRs between the classic and novel two-stage strategies in sensitivity analysis 3. The blue and red solid curves indicate the ERRs from the classic and novel two-stage strategies, respectively.

### 2.4 Sensitivity analysis 4

The centered knots are set as 10% and 30% quantile temperatures, i.e., -2.0 and 8.4 ℃. Compared to the results in the main analysis, Hainan and Guangdong province, where the temperature range is higher than the 30% overall quantile temperature 8.4 ℃, obtain much wide 95% confidence intervals within the observed temperature ranges, resulting the loss of statistical power.


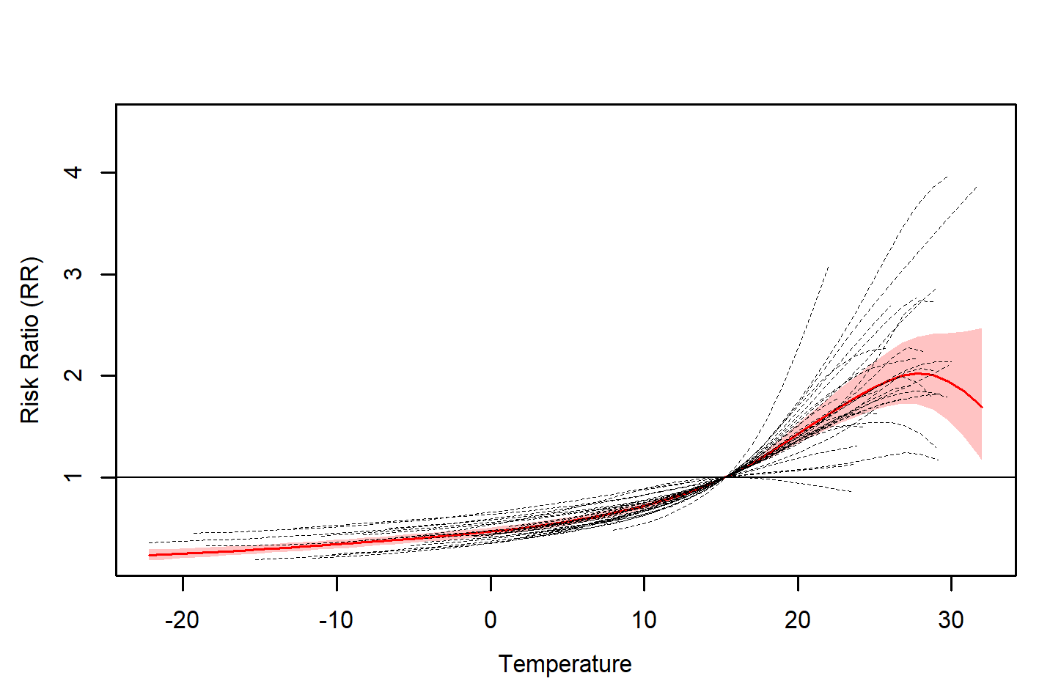


Fig J. The curves of pooled average and province-specific BD-temperature ERRs in sensitivity analysis 4. The red solid line is the average ERR and the shade is 95% confidence interval. The dashed lines are the province-specific ERRs within their corresponding temperature ranges.


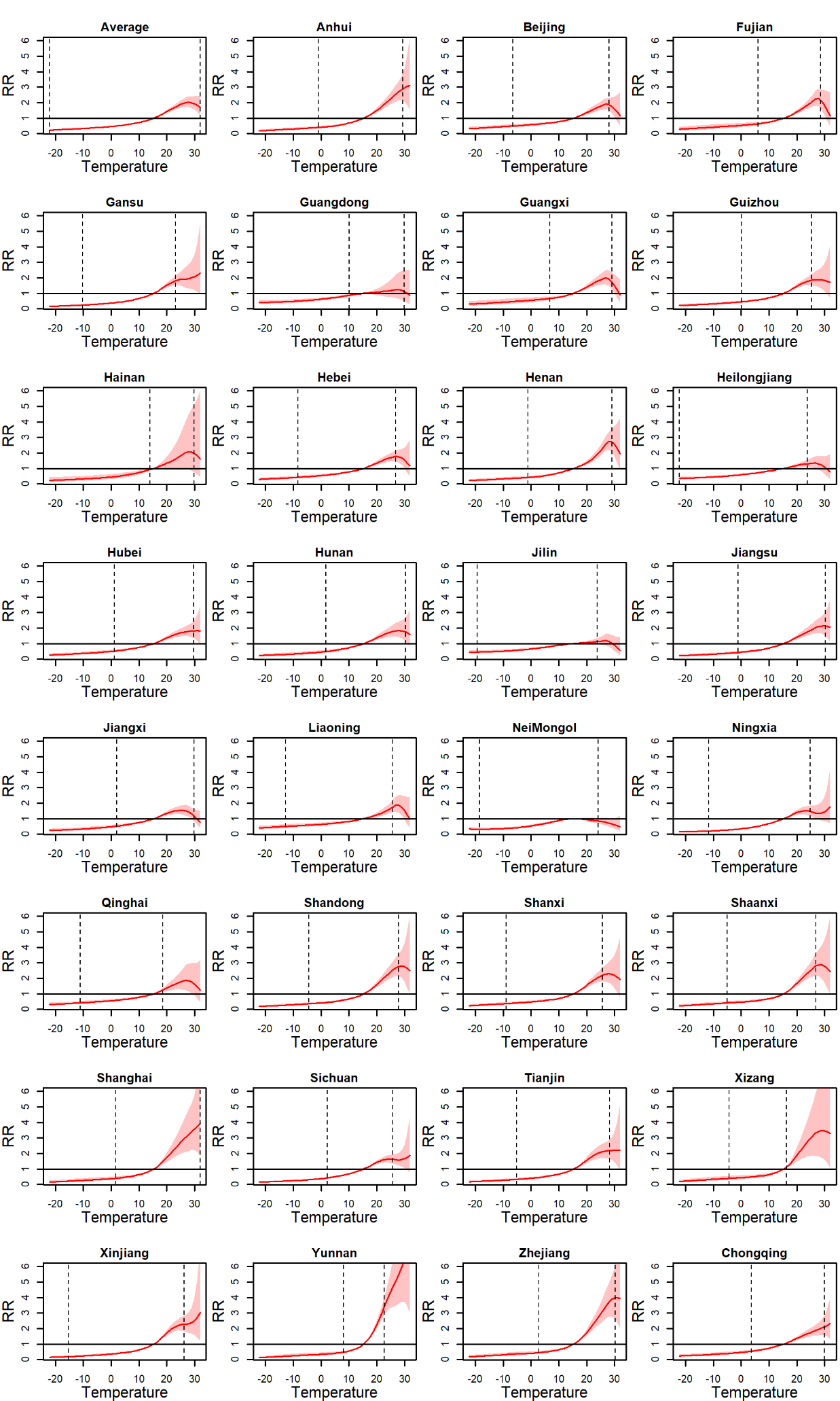


Fig K. The pooled average and province-specific ERRs in the overall temperature ranges in sensitivity analysis 4. The vertical dashed lines indicate the bounds of observed temperature in the corresponding province.


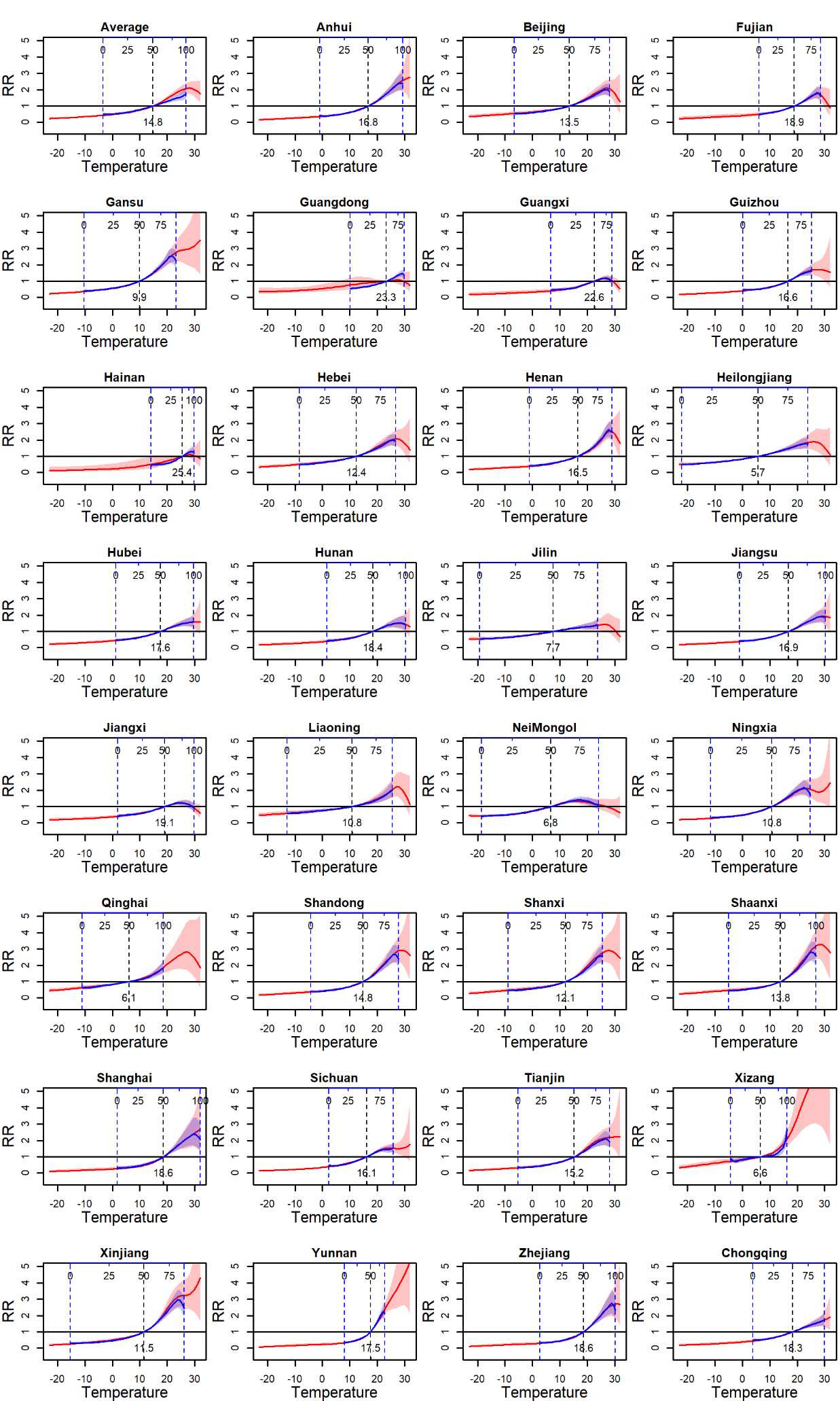


Fig L. The comparison of ERRs between the classic and novel two-stage strategies in sensitivity analysis 4. The blue and red solid curves indicate the ERRs from the classic and novel two-stage strategies, respectively.

### 2.5 Sensitivity analysis 5

We excluded the sunshine duration for a sensitivity analysis.


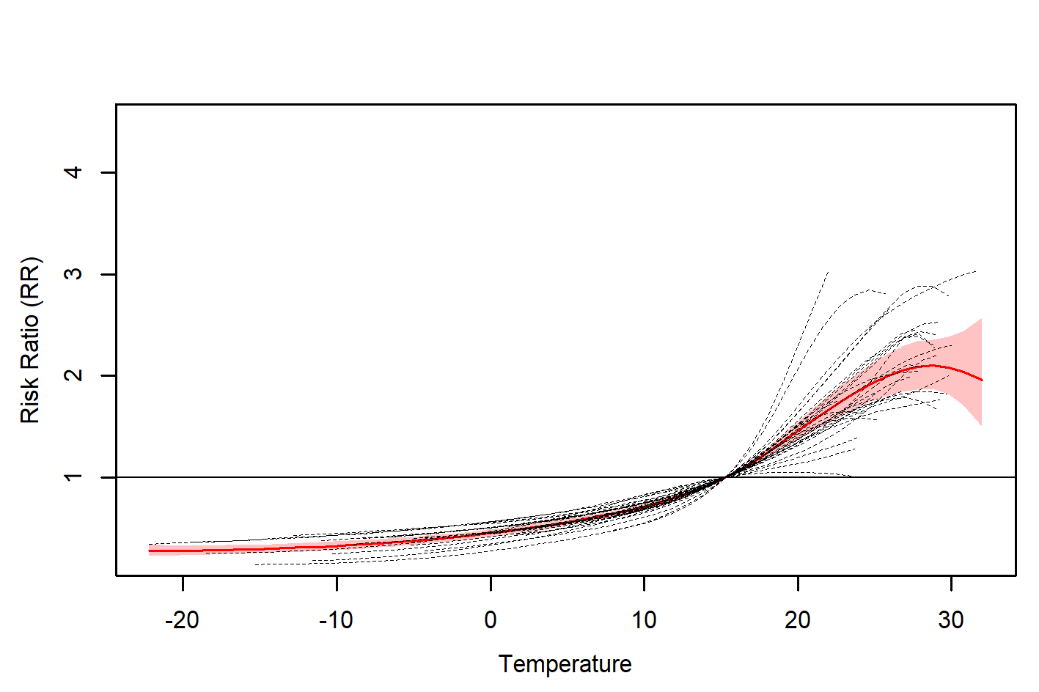


Fig M. The curves of pooled average and province-specific BD-temperature ERRs in sensitivity analysis 5. The red solid line is the average ERR and the shade is 95% confidence interval. The dashed lines are the province-specific ERRs within their corresponding temperature ranges.


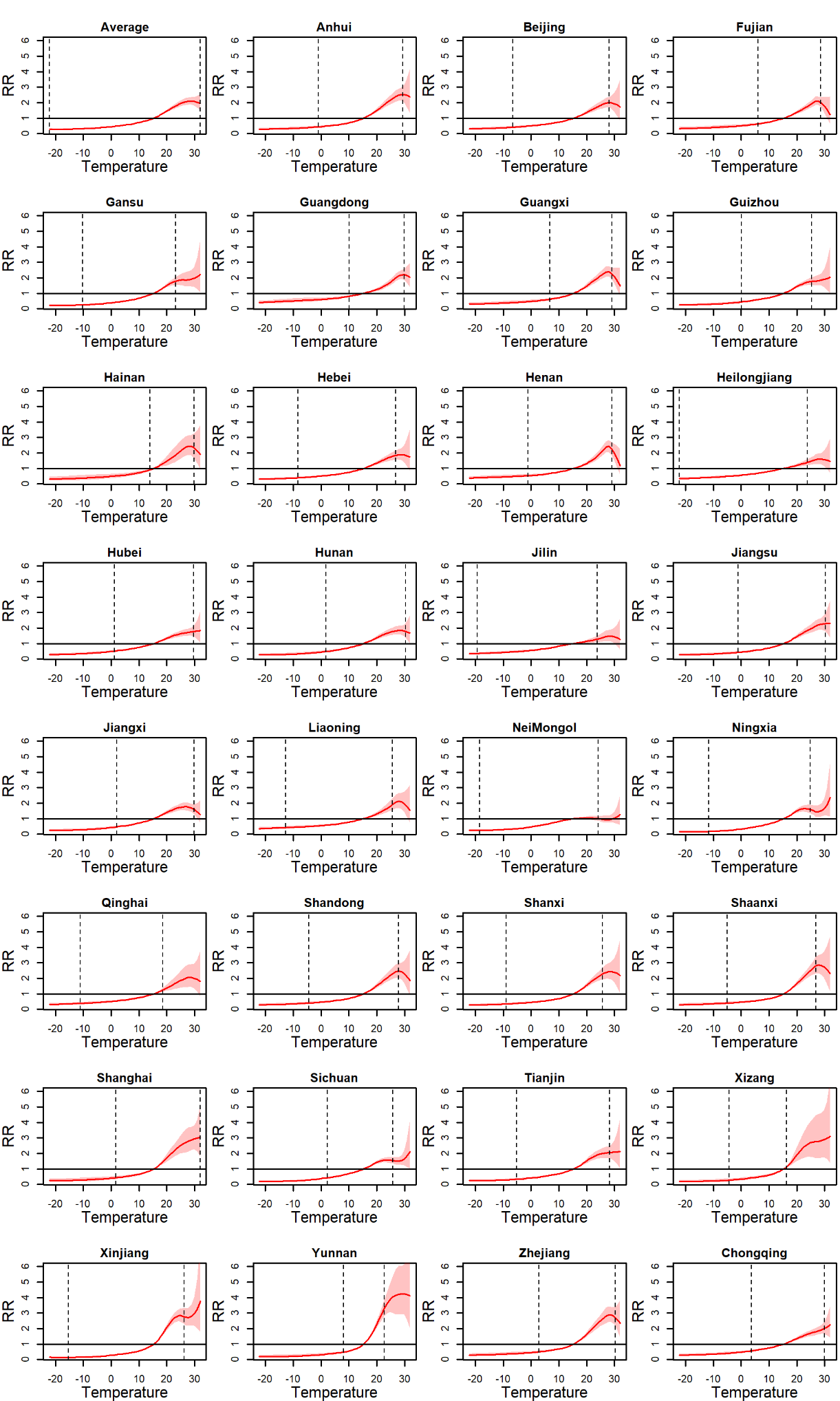


Fig N. The pooled average and province-specific ERRs in the overall temperature ranges in sensitivity analysis 5. The vertical dashed lines indicate the bounds of observed temperature in the corresponding province.

## 3. Comparison of BD-temperature ERRs on the original scale between the TS-based and B-spline-based strategies

The B-spline-based strategy is mentioned in the last part of Section 2.1 in the main text, which suffers from the mixing of different information and has never been applied in practice. Without statistical rationality, we also used the B-spline-based strategy to characterize the BD-temperature ERRs on the original scale only for providing a risk illustration about mistakenly using the B-spline-based strategy.


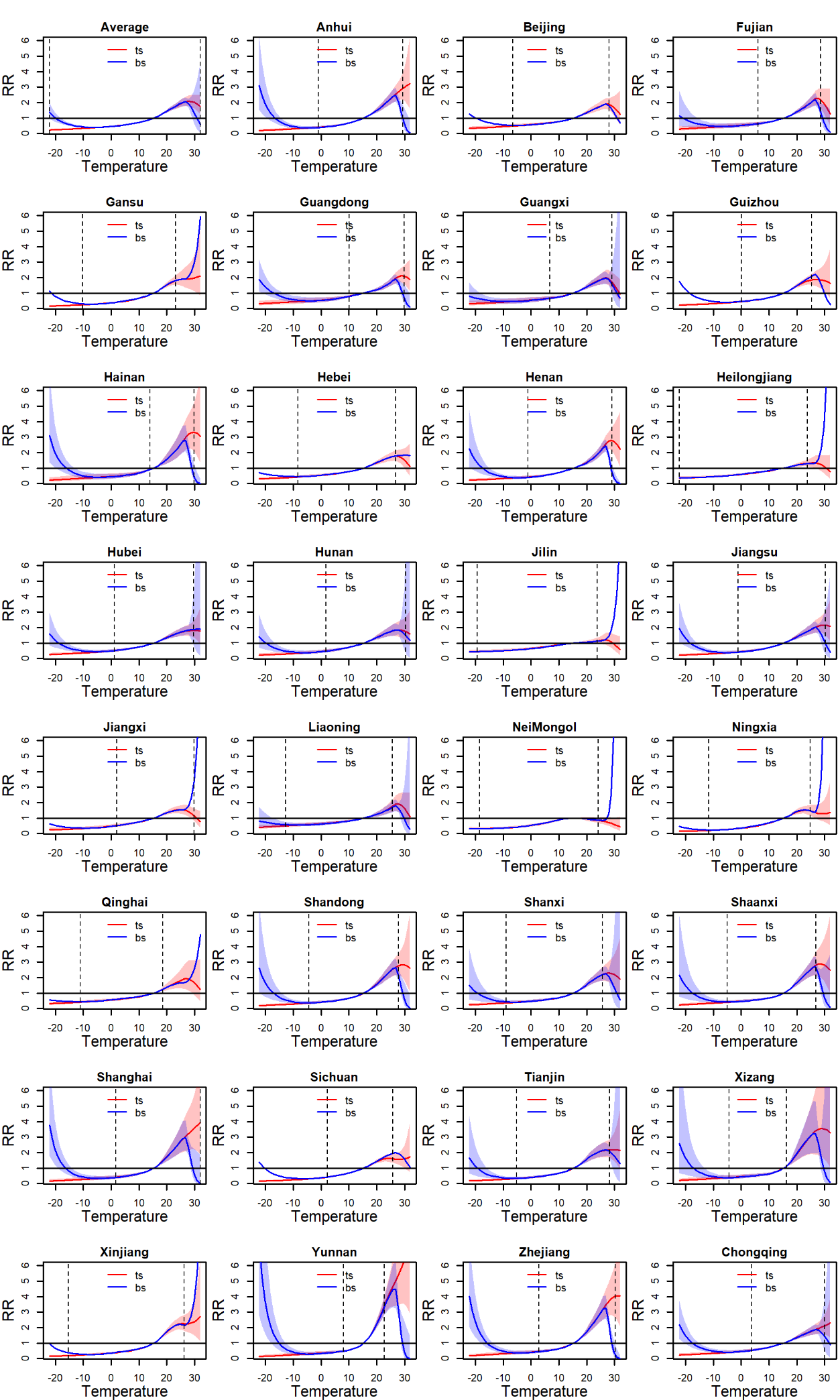


Fig O. The pooled average and province-specific ERRs on the original scale within the overall temperature range using the TS-based and B-spline-based strategies. The vertical dashed lines indicate the bounds of temperature in the corresponding province. The overall median temperature 15.3 ℃ is the reference.

## 4. A multi-region example with slightly different exposure ranges: comparison of ERRs on the original scale between the two strategies

Previous studies have shown that the TS has a good ability to fit nonlinear associations, but it is rarely used in environmental epidemiology. Therefore, we also used a multi-region study with slightly different exposure ranges to compare the two strategies in characterizing the ERRs on the original scale. This example was based on the work by Gasparini et al. via <https://github.com/gasparrini/2012_gasparrini_StatMed_Rcodedata>[1], which has been used to illustrate the classic two-stage strategy. This dataset included the time series data of daily temperatures and death counts for 10 regions in England and Wales during 1993-2006. In the classic strategy, the GAM in the first stage was constructed based on B-splines. The degree of the polynomial was set as 2 and the knots were set as 5%, 35%, 65%, and 95% quantile temperatures across all the region-level daily temperatures. To enhance the comparison, the same parameters were also set for TS in the novel strategies. The knots and temperature ranges are illustrated in Fig Pa.

The comparison of ERRs on the original scale between the two strategies is presented in Fig Pb. Both strategies could obtain region-specific ERRs outside the observed temperature ranges. The point estimations of ERRs and the 95% confidence intervals were almost the same between the two strategies, which suggested that the TS-based and the B-splines-based strategies have similar abilities in characterizing the nonlinear ERRs. We also changed the degree of the polynomial from 2 to 3, and the results remain identical.


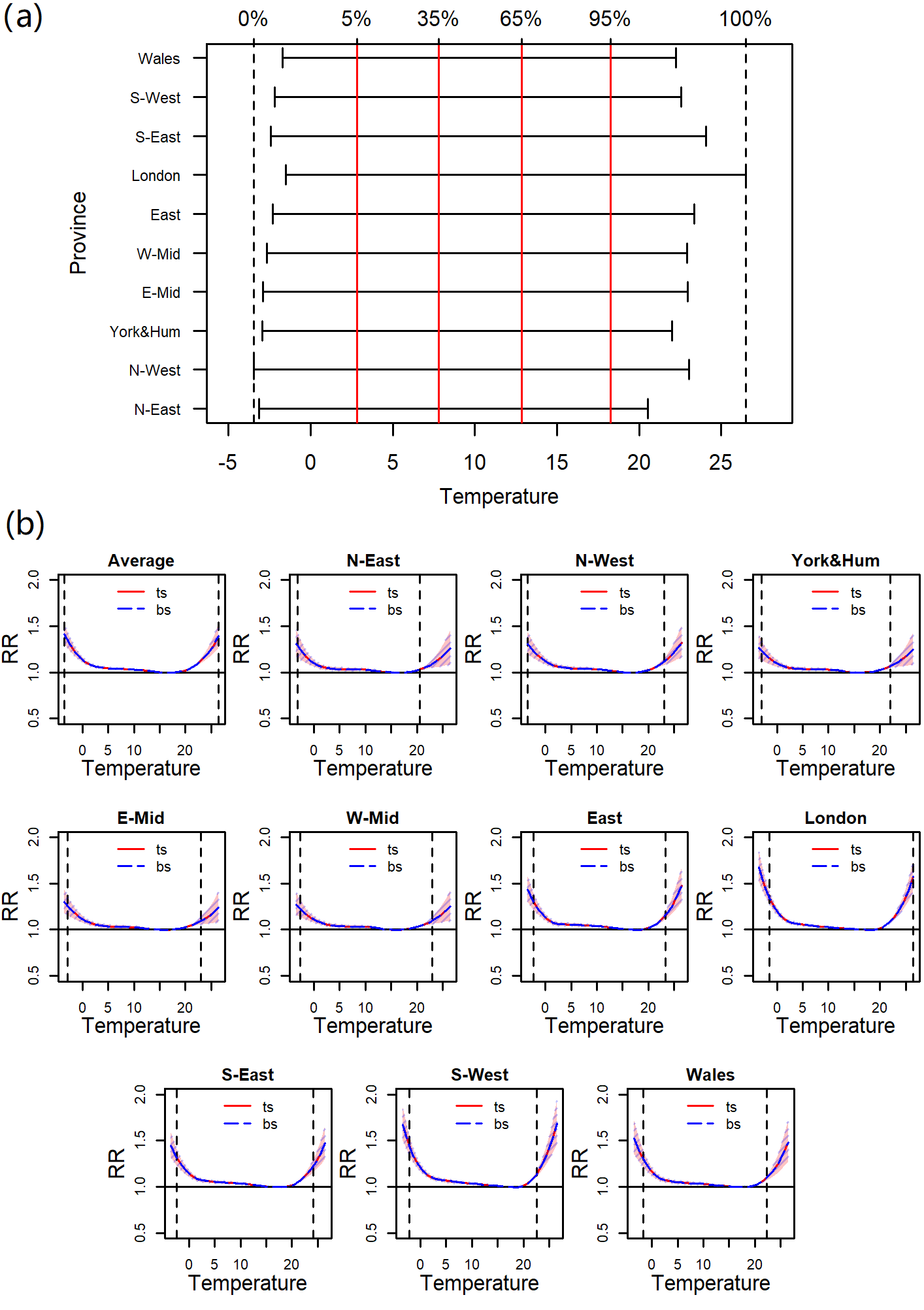


Fig P**.** Comparison of ERRs on the original scale between the TS-based and B-spline-based strategies in a multi-region study with slightly different exposure ranges (degree of the polynomial was set as 2). The top panel presents the temperature ranges in different regions. The bottom panel is the comparison of ERRs on the original scale, where ‘ts’ indicates the TS-based strategy and ‘bs’ indicates the B-spline-based strategy.

**Sensitivity analysis for changing degree of polynomial as 3.**


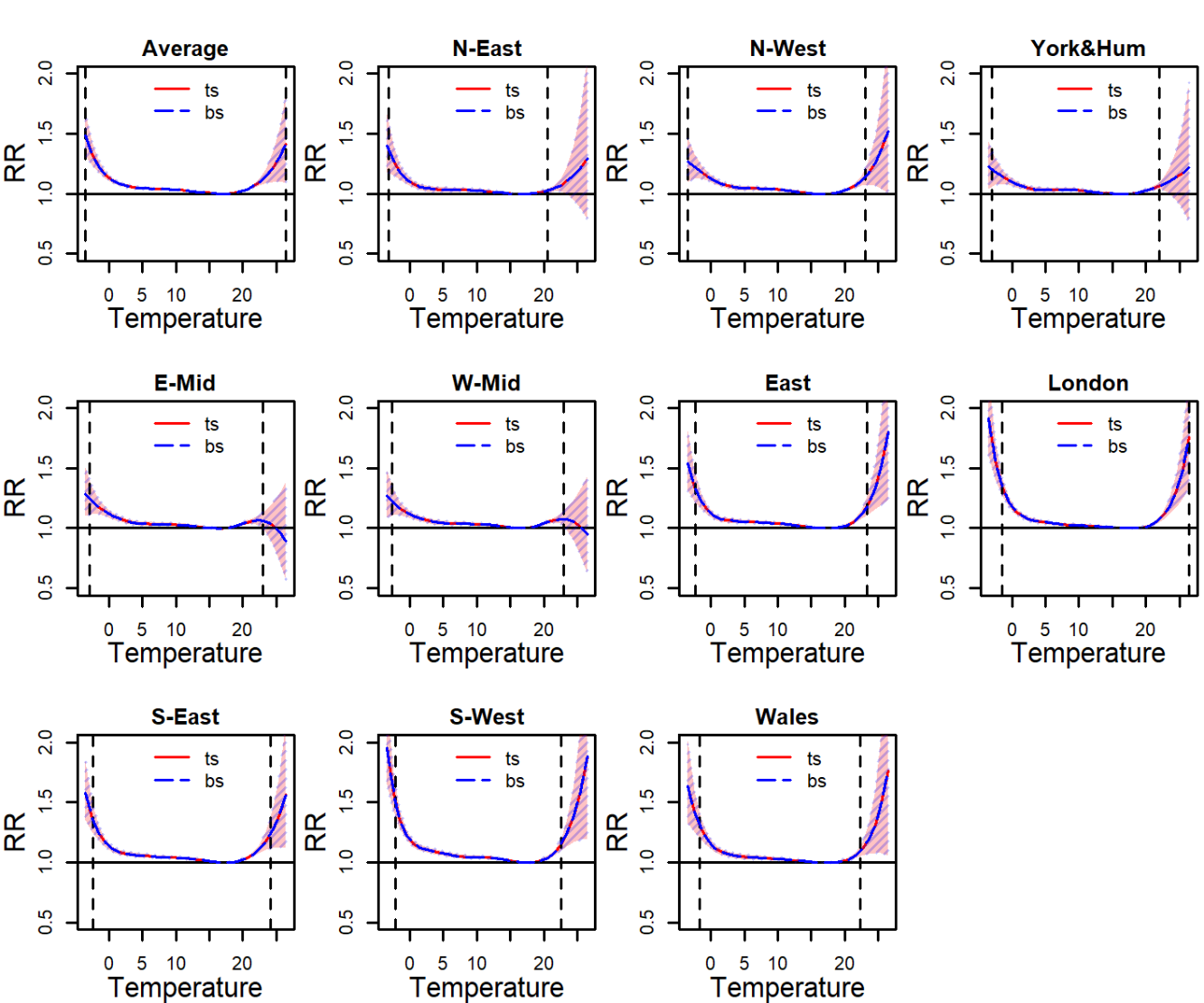


Fig Q. Comparison of ERRs on the original scale between the TS-based and B-spline-based strategies in a multi-region study with slightly different exposure ranges (degree of the polynomial was set as 3). ‘ts’ indicates the TS-based novel strategy and ‘bs’ indicates the B-splines-based classic strategy.

**Comparing the natural-splines-based classic strategy with the TS-based novel strategy.**


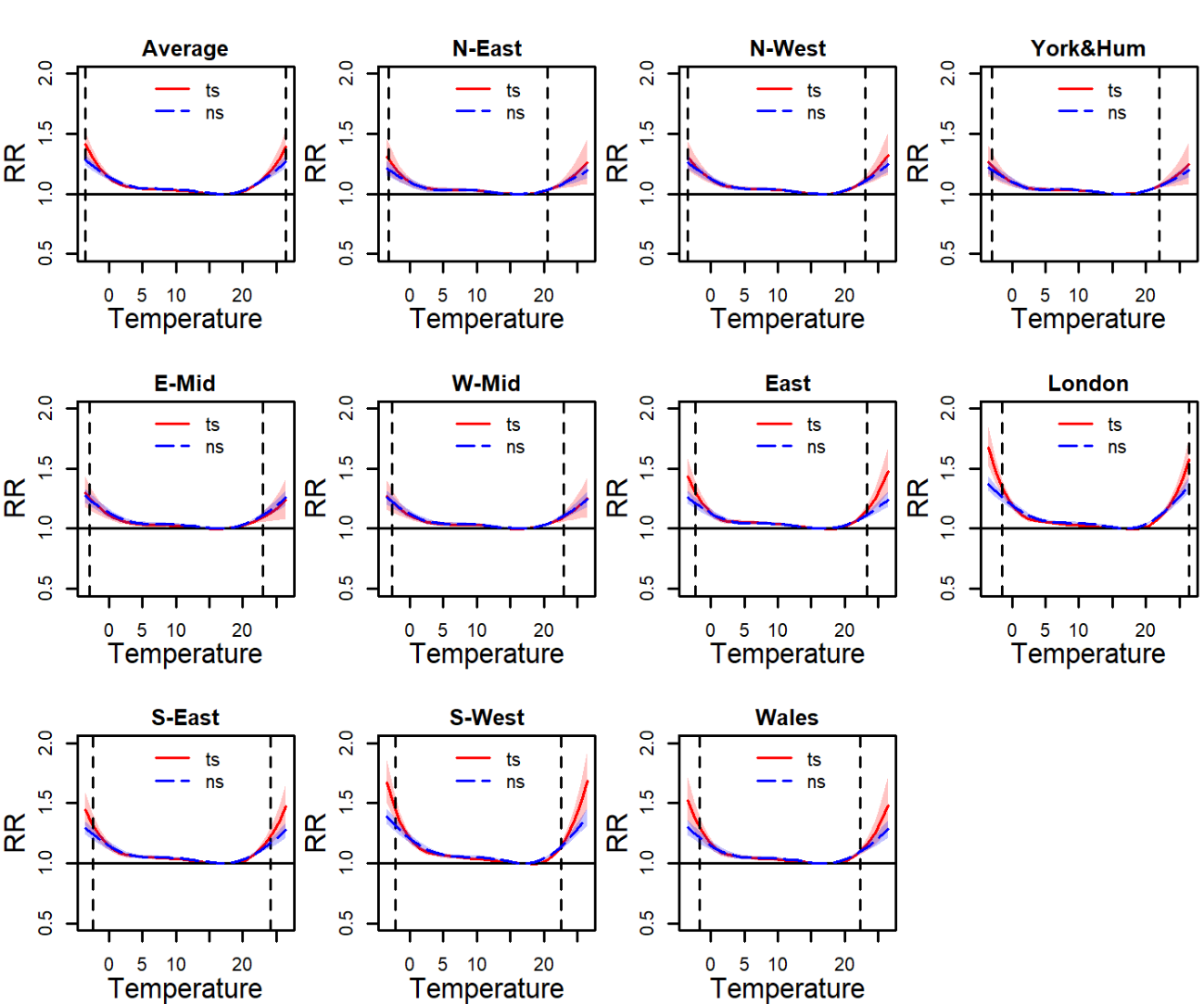


Fig R. Comparison of ERRs on the original scale between the TS-based and natural-spline-based strategies in a multi-region study with slightly different exposure ranges. ‘ts’ indicates the TS-based novel strategy and ‘ns’ indicates the natural-splines-based classic strategy.

**Reference**

1. Gasparrini A, Armstrong B, Kenward MG. Multivariate meta-analysis for non-linear and other multi-parameter associations. Stat Med. 2012;31(29):3821-39.
