# Supplementary material for: Inverted U-shaped association between bacillary dysentery and temperature: A new finding using a novel two-stage strategy in multi-region studies
Source: PLoS Negl Trop Dis. 2023 Nov 17;17(11):e0011771. doi: 10.1371/journal.pntd.0011771 (PMC10691710; doi:10.1371/journal.pntd.0011771)
Supplement: Attachment — Submitted filename: Response to reviewers.docx [file pntd.0011771.s002.docx]

# General reply

Many thanks to the referees for encouraging our work and giving useful comments. We have tried our best to revise the manuscript according to your kind and constructive comments and suggestions. The detailed revisions were marked via the Microsoft word track changes mode and has been uploaded as “Revised manuscript with changes highlighted.docx”. We sincerely hope that this revised manuscript has addressed all your comments and suggestions.

# Point-to-point replies to technical comments

## Editors:

The Authors are expected to address all the criticisms by all Reviewers. In particular, please assess the impact of spatial confounders and spatial heterogeneity on the results (Reviewers #1 and #3), provide justification for the extrapolation beyond the local temperature range and select the best model (Reviewer #2).

**Response to comments**

Thanks for the valuable comments. In the revised version, we have made a further anlaysis regarding the spatial confounders and spatial heterogeneity on the results, and provide the justification for the extrapolation beyond the local temperature range and select the best model. The detailed responses to these comments have been provided in the responses to the reviewers.

In additional to these comments, please address:

1. Please specify clearly the confounders in the Methods and provide the rationale

**Response to comments**

Thanks for the comments. Because previous studies have demonstrated that relative humidity and sunshine duration are two important factors which affect the bacillary dysentery (BD) incidence (Li et al. 2015 and Zhao et al 2021), we adjusted the two factors as potential confounders in the first-stage model. In addition, the longer-trend is also adjusted for obtaining a more stable result because the BD cases substantially decreases over time in China (Fig R1). According to the comments, we have provided a clearer explanation about the confounders in our methods, seen in lines 250-254 and 283-285 of the revised version. In addition, we also made a sensitivity analysis by excluding the sunshine duration according to the reviewer #2’s comment. The results remain stable, seen in the Fig S13-14 of the supplementary materials.

Fig R1. The variation trend of the number of BD cases in mainland China.

**Reference**:

Zhao R, Gao Q, Hao Q, et al. The exposure-response association between humidex and bacillary dysentery: A two-stage time series analysis of 316 cities in mainland China. Sci Total Environ. 2021;797:148840.

Li ZJ, Zhang XJ, Hou XX, et al. Nonlinear and threshold of the association between meteorological factors and bacillary dysentery in Beijing, China. Epidemiol Infect. 2015;143(16):3510-9.

2. Data should be described in the Methods

**Response to comments**

According to the editor’s comment, we have described the data in the Methods Section of the revised version.

3. Extrapolation was carried out sometimes >20 degrees outside the normal monthly average over 14 years. This is outside the expected range even with extreme weather.

**Response to comments**

Thanks for the comments. This extrapolation is derived from borrowing information from other provinces where the values of temperature are observed in the extrapolation range. Taking Guangxi Province as an example (seen in Fig R2), the observed temperature range is 7-29 ℃, and the extrapolated association is up to -22 ℃. Because all the other provinces presented a decreasing RR at low temperature, suggesting a stable epidemiological mechanism, the extrapolation RRs with narrow confidence intervals are still believable even outside the expected temperature range. We also acknowledged that the extrapolation for extremely low temperatures have a limited public health importance when the monthly average temperatures is used, but if one investigated the associations between daily temperature and BD incidence, the expected range may be expanded due to the larger fluctuation in daily temperatures. In such case, the extrapolation of our method would deserve more public health importance. According to comments, we have made a discussion about the public health importance of extrapolation, seen in lines 481-491 of the revised version.

Fig R2. The estimated association in Guanxi Province. The dashed vertical lines indicate the range of observed temperature.

4. Fig 5 shows a general pattern with peak at high temperature, however sometimes the extrapolated RR went over 5 which is higher than all predicted RR (e.g., Yunnan, Xizang) within the realized temperature range

**Response to comments**

Thanks for the comments. Seen in Fig R3, although Yunan and Xizang presented a high RR within the realized temperature range, but the confidence intervals are much wide especially at high temperatures, suggesting that the extremely high RR may not be believable at high temperatures. In contrast, the extrapolation RRs at low temperatures are believable due to the narrow confidence intervals. Unlike in other provinces, in Yunnan and Shanghai Province the association curves seem not to have a decrease tendency at high temperature, the reason may be complicated, such as 1) there is no enough power finding the decrease tendency due to the wide confidence interval at high temperatures, 2) a particular potential epidemiological mechanism may really lead to the continuously increasing RR over temperature. Further research based on individual data or daily-incidence is deserved for exploring the reasons. According to the comments, we made a further explanation about this issue in lines 304-308 of the revised version.

Fig R3. The association curves in Yunnan, Xizang, and Shanghai.

5. Different models were fitted and showed different results. The authors also present the data and fitting to allow further assessment.

**Response to comments**

Thanks for the comments. To allow further assessment about our proposed method, we have provided the data and R codes via <https://github.com/winkey1230/TS-based-strategy>. In the data section, we also supplemented the descriptive analysis about temperatures and BD incidences in the time dimension. The fitting indices are not statistically comparable between the TS-based method and B-splines-based method because the two methods have different research targets, i.e., the former is to investigate the association on original temperature scale and the latter is to investigate the association on relative temperature scale. Alternatively, we evaluated the two methods from epidemiological perspectives. In multi-region studies where the exposure ranges are largely different between regions, the advantages of the TS-based method over the B-splines-based method are 1) obtaining more rational epidemiological explanation, 2) investigating the association on original temperature scale, which provided more intuitive epidemiological implication, 3) having the extrapolation ability to some extent.

6. Fig 6, the estimates based on B-spline usually peaked earlier than that based on TS-spline. Which one is more reliable?

**Response to comments**

Thanks for the comments. In some provinces, the association curves based on B-spline usually peaked earlier than that based on TS-spline, but the average association curve across all the provinces did not present a turning point of decline. The reason is that the associations in provinces with high temperature ranges and the association in provinces with low temperature range are synthesized in the multivariate meta-analysis based on B-spline. Because the associations in low-temperature-range provinces (e.g., Liaoning) present an obviously monotone increasing trend, the average association were pooled to be more similar with the associations in low-temperature-range provinces than that in high-temperature-range provinces, thus a monotone increasing shape was examined in the average association. In a specific province, the association curves from the two methods are very similar within the observed temperature ranges. Even though a slight difference (e.g., the position of peak) is presented in some provinces, but the confidence interval suggests that this difference can be ignored, so it is difficult to judge which associations curve is more reliable. The advantages of TS-based associations curves were mainly reflected in the average association curve and the extrapolation ability. According to the comments, we have provided a more detailed explanation in lines 469-476 and 481-485 of the revised version.

In addition, the province-between heterogeneity of associations is relatively large ($I^{2} = 79\%$), which leads to that the association for a specific province is more determined by its own estimated association than by the borrowed associations from other provinces. Therefore, the association curves from the two methods are very similar within the observed temperature ranges. This similarity also suggest that TS and B-spines have comparable ability to characterize the nonlinear association in a single region study.

7. Fig 7 shows a U-shape relationship. Consider the temperature range, the results for low temperature is very different from Fig 5 & 6. How to reconcile these results?

**Response to comments**

We apologized for our unclear presentation. To demonstrate that the TS-based method also has a good performance in multi-region studies where the exposure variables present similar ranges, we used an additional public dataset to compare the TS-based method with the B-splines-based method. This additional dataset is regard to the association between temperature and mortality. Therefore, the results in Fig 7 are unrelated to those from Fig 5 & 6. To avoid confusion and make a more focus on the BD-temperature associations, we have moved the temperature-mortality association into supplementary materials.

8. Further, lowest risk was achieved at around 20 degrees (Fig 7), while moderate to high risk was achieved at the same temperature in Fig 5 & 6. How to reconcile these results?

**Response to comments**

We apologized for our unclear presentation. To demonstrate that the TS-based method also has a good performance in multi-region studies where the exposure variables present similar ranges, we used an additional public dataset to compare the TS-based method with the B-splines-based method. This additional dataset is regard to the association between temperature and mortality. Therefore, the results in Fig 7 are unrelated to those from Fig 5 & 6. To avoid confusion and make a more focus on the BD-temperature associations, we have moved the temperature-mortality association into supplementary materials.

## Reviewer #1:

Temperature stands as a crucial factor influencing BD incidence. Within the study, a two-stage strategy was proposed to explore the correlation between monthly temperature and BD incidence concerning the original temperature data. The study revealed a U-inverted shape in the average temperature-BD association. The manuscript is well-written; nevertheless, certain key issues need addressing before publication. The following points should be considered:

1. While numerous previous studies have documented a non-linear association between BD and climate factors, it is essential to provide a detailed explanation of the contribution of this study.

**Response to comments**

Thanks for the good comments. In a multi-region study where the ranges of exposure variable present largely difference between regions, the classic GAM-meta strategy only can explore the exposure-response association on relative exposure scale, which has been well demonstrated in Gasparini et al.’s work (2021). Due to the limitation of the classic strategy, previous nationwide multi-region studies only can characterize the temperature-BD association in relative temperature scale, which showed that BD incidence is positively associated with the relative temperature (Liu et al., 2020). This positive association does not conform to the laboratory knowledge that both high and low temperature interfere with the survival of bacteria. Furthermore, the obtained association on relative temperature scale also limits the intuition of epidemiological meanings. Therefore, we proposed a novel strategy to investigate the temperature-BD association on original temperature scale for a more intuitive understanding. Using the novel strategy, on average, an inverted U-shaped association between temperature and BD risk was observed in China, which conforms more to the laboratory knowledge. Furtherly, according to the province-specific association curves and the properties of meta-analysis, we explained the reason why the classic and novel strategies obtain different results. Our finding provided a better understanding regarding the temperature-BD association and may help design a more rational temperature-related public health policy in BD prevention and control.

In addition, compared to the classic strategy, the novel strategy can extrapolate the province-specific association outside the observed temperature ranges by utilizing information from other provinces, which is meaningful considering the frequent incidences of extreme temperatures. With numerous nationwide datasets where the ranges of exposure variable present largely difference between regions, the proposed strategy would have a wide application value.

To enhance the readability and quality of our work, according to the reviewer’s comment, we have given a more detailed explanation about the contribution of our study, seen in lines 446-454, 461-466, and 478-491.

**Reference**

Gasparrini, A., B. Armstrong, and M.G. Kenward, Multivariate meta-analysis for non-linear and other multi-parameter associations. Stat Med, 2012. 31(29): p. 3821-39.

Liu, Z., M.X. Tong, J. Xiang, et al., *Daily temperature and bacillary dysentery: estimated effects, attributable risks, and future disease burden in 316 Chinese cities.* Environmental health perspectives, 2020. **128**(5): p. 057008.

2. Additionally, the confidence interval for the association between BD and temperature in high values appears considerably large, potentially challenging the validity of the U-inverted shape conclusion. The reviewer suggests investigating potential spatial confounding of impact factors and recommends exploring the spatial heterogeneity of their association, such as by climate zone.

**Response to comments**

Thanks for the constructive comments. According to the review’s comments, we have made further analysis about the potential factors which may affect the temperature-BD associations and also explored the spatial heterogeneity between different natural zones.

Specifically, based on the estimated province-specific associations in the first stage, we incorporated a series of predictors, such as rainfall, relative humidity, poverty, education, GDP and so on, into the multivariate meta-regression to test the potential effect modifiers which may impact the temperature-BD associations. The poverty is measured by the proportion of people whose family incomes are below the lowest living standard and the education is measured by the number of pupils per teacher. More detailed information of predictors can be seen in Section 1.1 of the supplementary materials. Due to their significant spatial dependences, the incorporated predictors can control for the spatial confounding in the multivariate meta-regression to some extent. In addition, we also used the multivariate conditional meta autoregression (Wang et al. 2023) to test the residual spatial dependence in the meta-regression and results showed that there is no significant residual spatial dependence, suggesting that the spatial confounding was well controlled.

Seen in Table R1, the I^2^ statistics showed that a considerable heterogeneity exists between provinces. The meta-regressions only with a predictor identified the poverty and rainfall as significant modifier effects (P < 0.05). The P values of relative humidity and education were closed to 0.05. After using an AIC-based forward method to select variables, only poverty and education were included into the multiple meta-regression with P < 0.001, suggesting that poverty and education were two important factors of affecting the temperature-BD associations, which conforms to the expectation since BD is a poverty-related infectious diseases. Furtherly, based on the multiple meta-regression, we plotted the temperature-BD associations at high/low poverty and good/poor education. Shown in Fig R4, the four association curves presented a U-inverted shape; especially at low poverty and good education, the U-inverted shape is more obvious. For the associations at high poverty and poor education, the risk of BD rose as temperature increased until temperature is above 29 ℃. In addition, by comparing the slopes in the association curves, the BD incidence at good education seems to be less sensitive to temperature than that at poor education. The reason may be that people with good education have more temperature-related consciousness of protection, thus reducing the impact of temperature on BD incidence.

According to the climate and geography, we also divided the study region into 7 zones and plotted the average association in each zone. Seen in Fig R5, because the subgroup analysis blocks the information borrowing between zones, we obtained the association curve only within the observed temperature range for a specific zone. Overall, the BD incidence rose as temperature increased in all zones, but when temperature was above 28-30 ℃, apart from the southwest of China, the BD incidence no longer rose and even started to decline especially in the east of China. In the northeast, the association curve presented a flatter slope, suggesting that the BD incidence is less sensitive to temperature; we did not obtain its association at a high temperature (>25℃) due to the observed temperature ranges less than 25℃.

**Reference**

Wang W, Liao F, Yin F, Ma Y. Mapping the short-term exposure-response relationships between environmental factors and health outcomes and identifying the causes of heterogeneity: A multivariate-conditional-meta-autoregression-based two-stage strategy. Spatial Statistics. 2022:100720.

Table R1. The identification of effect modifiers and heterogeneity tests in multivariate meta-regressions.

| Predictors ^a^ | AIC |  | LR ^b^ |  |  | Q test ^c^ | | | I^2^ |
| --- | --- | --- | --- | --- | --- | --- | --- | --- | --- |
|  |  | Stat | df | *P* |  | Stat | df | *P* |  |
| Intercept | -239.6 | - | - | - |  | 875.1 | 180 | < 0.001 | 79.4 |
| Rainfall | -241.8 | 14.2 | 6 | 0.028 |  | 802.7 | 174 | < 0.001 | 78.3 |
| Humidity | -238.7 | 11.1 | 6 | 0.086 |  | 820.6 | 174 | < 0.001 | 78.8 |
| Sunshine | -237.4 | 9.8 | 6 | 0.135 |  | 813.1 | 174 | < 0.001 | 78.6 |
| GDP | -236.2 | 8.5 | 6 | 0.203 |  | 851.7 | 174 | < 0.001 | 79.6 |
| Wage | -234.1 | 6.4 | 6 | 0.375 |  | 860.1 | 174 | < 0.001 | 79.8 |
| Urbanization | -235.1 | 7.4 | 6 | 0.284 |  | 850.2 | 174 | < 0.001 | 79.5 |
| Poverty | -247.9 | 20.2 | 6 | 0.003 |  | 769.7 | 174 | < 0.001 | 77.4 |
| Education | -239.7 | 12 | 6 | 0.062 |  | 768.4 | 174 | < 0.001 | 77.4 |
| Doctors | -234.1 | 6.4 | 6 | 0.376 |  | 837.6 | 174 | < 0.001 | 79.2 |
| Health worker | -233.4 | 5.7 | 6 | 0.452 |  | 851.9 | 174 | < 0.001 | 79.6 |
| Beds | -235.7 | 8 | 6 | 0.236 |  | 834.3 | 174 | < 0.001 | 79.1 |
| Poverty + education | -250.4 | 34.8 | 12 | < 0.001 |  | 693.1 | 168 | < 0.001 | 75.8 |

Note: ^a^ The detailed explanations for the predictors can be seen in the supplementary materials. Intercept indicates that the multivariate meta-regression includes only an intercept and no predictor. ^b^ The likelihood ratio test was used to test if a predictor significantly modifies the association. ^c^ The I^2^ statistic was used to measure the residual heterogeneity and Cochran Q test was used to test the residual heterogeneity.

Fig R4. The temperature-BD associations at different poverty and education levels. The association curves come from the multivariate meta-regression with poverty and education as predictors. The high and low poverties refer to the proportions of people whose family incomes are below the lowest living standard as 0.135 and 0.007, respectively. The good and poor educations refer to the number of pupils per teacher as 11 and 20, respectively.

Fig R5. The associations between temperature and BD incidence in the seven zones.

3. For a more comprehensive discussion, it is advisable to include more comparisons between this study and other relevant papers in the manuscript. This will help contextualize and enrich the findings, contributing to the overall understanding of the subject matter.

**Response to comments**

Thanks for the comments. In the revised version, we have further compared our study with previous studies to help understand our work. The further discussion can be seen in lines 476-480 and 488-491 of the revised version.

## Reviewer #2:

This manuscript looks at association between bacillary dysentery and temperature, and disparities between provinces in China. While this is an interesting study, I have significant concerns with the paper’s methodology. I believe that getting these methods points nailed down is critical for publishing in a high visibility place like PLOS Neglected Tropical Diseases.

1. Line 165: the sentence is not complete. the matrix composed of 1-5 and 1 is also “what” for natural splines?

**Response to comments**

We apologized for the uncomplete sentence. According to the reviewer’s comment, we have changed this sentence as “the matrix composed of $b_{1}$-$b_{5}$ and 1 is also singular for natural splines”, seen in lines 164.

2. Fig 1: please provide more information on the b1-b5 in the left panel and b1-b6 in the right panel. What does the term “b6” stand for?

**Response to comments**

Thanks for the good comments. In the left panel of Fig 1, b1-b5 indicate the five basis functions and their linear combination forms the natural spline with four knots (0.2, 0.4, 0.6, and 0.8) and two bounds (0 and 1). Similarly, in the right panel of Fig 1, b1-b6 indicate the six basis functions and their linear combination forms the B-spline function with four knots (0.2, 0.4, 0.6, and 0.8) and two bounds (0 and 1). The detailed mathematical expressions for these basis functions were complicated, which have been well presented in Wood et al.’s work (2006). According to the reviewer’s comments, we have provided a more detailed explanation to enhance the readability, seen in lines 177-180.

**Reference**

Wood SN. Generalized Additive Models: An Introduction With R. New York Springer; 2006. p. 249-307.

3. Method:

(1) The term "truncated polynomial splines" is confusing. As far as I understand, both B-splines and natural cubic splines are types of polynomial splines. What distinguishes proposed TS in the present study and other polynomial splines like B-spline and natural cubic spline?

**Response to comments**

We agree with the reviewer that B-splines and natural cubic splines are types of polynomial splines. Truncated polynomial splines (TS) are also type of polynomial splines. All the three splines can be used to characterize the nonlinear association. In a single region study, B-splines and natural splines can obtain good performance. However, in a multi-region study in which the exposure variable presents largely different ranges between regions, when one employs the commonly used meta-based two-stage strategy, B-splines and natural splines cannot characterize the exposure-response association on original exposure scale due to the possible singular matrices (Gasparrini et al. 2012). Alternatively, researchers can only obtain the associations on relative exposure scale (Gasparrini et al. 2012), but which may limit the intuition of epidemiological meanings and even lead to an unreasonable result in some cases. To address this issue, we proposed to use TS to characterize the nonlinear association in the two-stage strategy, when a large difference of exposure ranges is observed between regions.

The largest difference between TS and B-splines (or natural splines) is as follow. Given any knots, in TS each knot defines a basis variable by itself, thus the matrix composed of the non-zero basis variables is always non-singular and can avoid complete multicollinearity, which makes the meta-analysis works and valid in the second stage. However, in B-splines and natural splines, each basis variable was jointly defined by multiple knots (de Boor 1978 and Lancaster et al. 1986), thus the matrix composed of the non-zero basis variables is sometimes singular when the knots are outside the observed exposure range, which often occurs in multi-region studies with largely different exposure ranges. To help understanding, we gave an intuitive illustration example to present the difference between TS and B-splines (or natural splines) in suffering from singular matrices. The R codes for this example is available via <https://github.com/winkey1230/TS-based-strategy> (see the file “Basis functions for ns bs ts.R”).

To enhance the readability and quality of our paper, according to the reviewer’s comments, we make a further explanation about the difference between TS and B-splines (or natural splines) and our motivation of proposing TS, seen in lines 157-166 and 183-207 of the revised version.

**Reference**

Gasparrini A, Armstrong B, Kenward MG. Multivariate meta-analysis for non-linear and other multi-parameter associations. Stat Med. 2012;31(29):3821-39.

de Boor (1978). *A Practical Guide to Splines*. New York: Springer.

Lancaster, P. and K. Šalkauskas (1986). Curve and Surface Fitting: An Introduction. London: Academic Press.

(2) It is suggested to use smoothing splines rather than polynomial regression for the following reasons: First, smoothing splines yield a piecewise continuous function composed of many polynomials to model the dataset, while polynomial regression provides a single polynomial that models the entire dataset. Second, polynomial regression may not handle thresholds well, especially when certain values of the predictor strongly influence the response at a different value of the predictor (Nonlocal Behavior in Polynomial Regressions). However, thresholds are quite common in modeling the effect of temperature on health. Third, smoothing splines are better suited for extrapolation, even at extreme values. Has the proposed method addressed or resolved these issues? To demonstrate the superiority of the novel method over classic methods, the authors should provide additional tests and evidence supporting its better performance.

**Response to comments**

Thanks for the comments. We agree with the reviewer that smoothing splines, such as B-spline and natural splines, can obtain better performance than polynomial regression. However, in a multi-region study, when we need to obtain more stable associations and average association and to explore the region-between heterogeneity, a meta-analysis/regression is often constructed based on the region-specific associations from the first stage. In such case, if we used B-spline and natural splines to characterize the region-specific associations, the meta model would not work or lose meanings due to the possible singular matrices (or complete multicollinearity between basis variables) when the ranges of exposure variable present large difference between regions. Therefore, we alternatively proposed to use the truncated polynomial splines (TS) to address this issue, which can exactly avoid the trouble from singular matrices and complete multicollinearity between basis variables.

As the reviewer say that polynomial regression provides a single polynomial that models the entire dataset, which may not handle the thresholds well, thus we used the truncated polynomial splines (TS) rather than the polynomial regression. Like natural spline and B-splines, the TS is also a piecewise continuous function composed of many polynomials. In addition, we also limit the order of polynomials in TS as 2 or 3 to furtherly relieve the nonlocal behavior. Previous studies have demonstrated that the TS can obtain substantially better performance than the polynomial regression (Wood 2006).

In epidemiological studies, researchers rarely used the smoothing splines to extrapolate an association curve within an unobserved exposure range. However, in our work, this extrapolation for TS is not derived from the properties of smooth splines, but from the properties of meta-analysis by borrowing information. We used a simple example to explain the rationality underlying the extrapolation. Considering that the observed exposure range is 0-25 ℃ in region A and 0-30 ℃ in other regions, then we can use TS to directly estimate the association curves within 0-25 ℃ in region A and within 0-30 ℃ in other regions. Given the identical knots and bounds within the overall range (0-30 ℃) in all the regions, 1) the estimated parameters defining the association curves have the same mathematical explanation among regions and 2) the parameters related to the association curves within 25-30 ℃ would be missing in region A because the corresponding basis variables are constantly equal to 0. Then, by utilizing the similarity among regions, we can use meta-analysis to fill in the missing values in region A by borrowing the information from other regions. Therefore, the association in region A within 25-30 ℃ is extrapolated. Such extrapolation is based on the observation values in other regions and the region-between similarity. To enhance the readability, we gave a further explanation about the rationality of extrapolation for TS in lines 235-241 and 491-493 of the revised version.

In contrast, if B-splines and natural splines were employed, the basis variables related to the association curves within 25-30 ℃ would be completely multicollinear with other basis variables, which confuses the parameter estimations. In such case, if the meta-analysis is forcibly used in the second stage regardless of the rationality, the information borrowing can be interpreted as mixing variables with different meanings, which leads to a useless analysis. Seen in Fig R6, the association curves obtained by B-spline-based meta-analysis showed a quite odd shape, which violates the existing epidemiological evidence and is not acceptable. For the “additional tests” for supporting our method, mentioned by the reviewer, it is difficult to carry out a statistical comparison between B-spline-based and TS-based strategies. The reason is that the estimated parameters from B-splines in the first stage have different epidemiolocal meanings between regions, which leads to that any subsequent statistical model (e.g., meta model) cannot be constructed with rationality. In practices, when the multi-region study presents largely different exposure ranges, researchers often use the B-spline-based strategy to investigate the association on the relative exposure scale rather than the original exposure scale (Gasparrini et al. 2012). That is why we put the Fig R6 in the supplementary materials rather than in the main text.

In summary, in the context that there is no a valid method investigating exposure-response associations on the original exposure scale in a multi-region study with largely different exposure ranges, we proposed to use the TS-based strategy to address this issue. To enhance the readability, according to the reviewer’s comments, we provided a more detailed description about our method, seen in lines 182-234 of the revised version.

**Reference**

Wood SN. Generalized Additive Models: An Introduction With R. New York Springer; 2006.

Gasparrini A, Armstrong B, Kenward MG. Multivariate meta-analysis for non-linear and other multi-parameter associations. Stat Med. 2012;31(29):3821-39.

Fig R6. The pooled average and province-specific ERRs on the original scale within the overall temperature range using the TS-based and B-spline-based meta-analysis. The vertical dashed lines indicate the bounds of temperature in the corresponding province. The overall median temperature 15.3 ℃ is the reference.

(3) The authors should refrain from assessing the results of B-splines based on "common epidemiological sense." The same knot setting or degrees of freedom may yield different curves and it is not uncommon for b-spline with three knots (10, 50, 90) to generate linear. For example, Liang et al. (https://doi.org/10.1186/s12940-016-0166-4) applied a cubic b-spline with 5 df for the daily mean temperature and 4 df in the lag space and results represented different patterns of temperature-health effects. For Fig 13, statistical tests should be provided to determine which model offers the best fit.

**Response to comments**

Thanks for the reviewer’s comments. Both epidemiological and biological studies have demonstrated that an extremely low temperatures would suppress the BD incidence, which has been well acknowledged, so using the solid epidemiological evidence can help readers to understand the advantage of our proposed method in practical epidemiological applications. According to the reviewer’s comment, we have changed "common epidemiological sense" to “existing epidemiological evidence”, seen in lines 321.

We agree with the viewer that the same knot setting or degrees of freedom may yield different curves. In our results, given the same knot setting and degrees of freedom, Both B-splines and TS also generate different curves, seen in Fig 8 of the main text.

In Fig S13 (Fig S15 in the revised version), the TS-based strategy and B-splines-based strategy were used to characterize the associations on the original scale for a comparison. Because the non-zero basis variables for B-splines presented complete multicollinearity, the parameter estimation process in the first stage cannot be carried out. If one forcibly does by arbitrarily setting some of the parameters as missing, the remained estimated parameters would have different epidemiological meanings between regions because the multicollinear basis variables may be different between regions, which leads to that the meta-analysis cannot be constructed with statistical rationality. Furthermore, the estimated parameters from TS and B-splines are two different outcome variables in meta-analysis, which also leads to the lack of statistical comparability between the two models. In practical applications, when the multi-region study presents largely different exposure ranges, researchers often use the B-spline-based strategy to investigate the association on the relative exposure scale rather than the original exposure scale. That is why we put the Fig R6 in the supplementary materials rather than in the main text.

(4) It is not necessary for the knots and bounds to be identical to generate basis variables with the same mathematical meaning among regions. Researchers can build region-specific basis variables, and likelihood-ratio tests can be employed to select the best model for each region or province in the current study.

**Response to comments**

Thanks for the comments. In multi-regions studies, we aim to obtain the average association and more stable province-specific associations by borrowing information and to explore the heterogeneity of associations, thus the meta-analysis/regression is needed to model the region-specific associations from the first stage. In the first stage, if the knots and bounds were not identical, the basis variables would have different mathematic meanings (i.e., different mathematic expressions) between regions. In such case, the estimated parameters would have different epidemiological meanings. If the meta-analysis is forcibly constructed without rationality, the information borrowing can be interpreted as mixing apples and oranges, leading to that the meta-analysis is useless. In existing studies, when regions present similar exposure ranges, researchers often keep the knots and bounds to be identical on original exposure scale for investigating association on original exposure scale; when regions present largely different exposure ranges, researchers often keep the knots and bounds to be identical on relative exposure scale for investigating association on relative exposure scale. The necessary of the knots and bounds to be identical has well be presented in Gasparrini et al.’s work (2012).

**Reference**

Gasparrini, A., B. Armstrong, and M.G. Kenward, Multivariate meta-analysis for non-linear and other multi-parameter associations. Stat Med, 2012. 31(29): p. 3821-39.

(5) B-spline and natural cubic spline could also provide estimates on the original temperature scale. Can B-splines and natural cubic splines be truncated like what the authors did with polynomial regression? It would be helpful and meaningful to explore this possibility.

**Response to comments**

Thanks for the comments. B-splines, natural cubic splines, truncated polynomial splines (TS) are piecewise functions. The difference is their methods for constructing basis functions. Given the knots and bounds outside the observed exposure ranges, the non-zero basis variables from B-spine and natural cubic splines may be completely multicollinear, while the TS can avoid this trouble. Because B-splines and natural cubic splines are already piecewise functions based on the give knots and bounds, they cannot be furtherly truncated like the TS. Otherwise, the methods for constructing the basis functions would change radically, that is why we proposed to use TS.

(6) Why was sunshine duration included in the model? Could it explain part of the effects of temperature?

**Response to comments**

Thanks for bringing us into this concern. To some extent, sunshine duration is related to temperature, but it is not completely the part of temperature. For example, in Sichuan province where the main terrain is basin, sunshine duration is often short, but temperature is high, because the thick cloud can reduce heat dissipation and sunlight penetration. In Mongolia and Xinjiang province where the altitude is relatively high and over 1000m, although sunshine duration is long, but temperature is not high. Furtherly, we calculated the correlation between sunshine duration and temperature, which is relatively small (around 0.08). According to the reviewer’s comments, we excluded the sunshine duration from the model for a sensitivity analysis. The results remain stable, which is shown in the Fig S13-14 of the supplementary materials.

4. Fig 6: can the authors provide justification for the extrapolation of the curve to temperatures beyond the local temperature range?

**Response to comments**

We apologized for our unclear expressions for this justification. In our work, this extrapolation for TS is not derived from the properties of smooth splines, but from the properties of meta-analysis by borrowing information. Here, we used a simple example to explain the rationality underlying the extrapolation. Considering that the observed exposure range is 0-25 ℃ in region A and 0-30 ℃ in other regions, then we can use TS to directly estimate the association curves within 0-25 ℃ in region A and within 0-30 ℃ in other regions. Given the identical knots and bounds within the overall range (0-30 ℃) in all the regions, 1) the estimated parameters defining the association curves have the same mathematical explanation among regions and 2) the parameters related to the association curves within 25-30 ℃ would be missing in region A because the corresponding basis variables are constantly equal to 0. Then, by utilizing the similarity among regions, we can use meta-analysis to fill in the missing values in region A by borrowing information from other regions. Subsequently, the association in region A within 25-30 ℃ is extrapolated. Such extrapolation is based on the observation values in other regions and the region-between similarity. To enhance the readability, we gave a further explanation about the rationality of extrapolation for TS in lines 235-241 and 491-493 of the revised version.

5. Fig S14-15 are not referenced in the main text.

**Response to comments**

We apologized for our unstandardized presentation. In the revised version, we supplemented the information in the main text, seen in lines 316, 320 and 443 of the revised version.

6. Lines 275-276: The long-term trend should be characterized by a natural cubic spline with a df of 6 "per year." The phrase "per year" is missing in this statement.

**Response to comments**

Thanks for the comments. Due to the data availability, in our case study, we investigated the associations between monthly temperature and BD incidence. So, the df of long-term trend is 6 for all the studied time span, which is selected by minimizing the value of AIC, shown in Fig R7. The sensitivity analysis for df of 4 and 8 remained stable, seen in Fig S4-S9 of the supplementary materials.

Fig R7. The values of AIC at different dfs for the long-term trend.

## Reviewer #3:

1.	Spline seems too strict to fit the volatile real data.

**Response to comments**

Thanks for the comments. Because the real association between environment and human health is determined by one or several potential epidemiological mechanisms, the association curves in epidemiological studies usually present no complicated fluctuation, otherwise, the epidemiolocal explanation is unavailable. So, splines are enough flexible for charactering the nonlinear associations between environment and human health, which have commonly used in epidemiological studies (Chen et al. 2018, Vicedo-Cabrera et al.2021, Liu et al. 2019, Gasparrini et al. 2015).

**Reference**

Chen R, Yin P, Wang L, et al. Association between ambient temperature and mortality risk and burden: time series study in 272 main Chinese cities. *Bmj.* 2018;363:k4306.

Vicedo-Cabrera AM, Scovronick N, Sera F, et al. The burden of heat-related mortality attributable to recent human-induced climate change. *Nature climate change.* 2021;11(6):492-500.

Liu C, Chen R, Sera F, et al. Ambient Particulate Air Pollution and Daily Mortality in 652 Cities. *The New England journal of medicine.* 2019;381(8):705-715.

Gasparrini A, Armstrong B, Kenward MG. Multivariate meta-analysis for non-linear and other multi-parameter associations. *Statistics in medicine.* 2012;31(29):3821-3839.

2.	Create a map of the disease and overlay it with relevant variates.

**Response to comments**

According to the comments, in the revised version, we have supplemented the descriptive analysis about the spatial distributions of temperature and BD incidence, seen in Section 2.3 and Fig 3 of the main text.

3.	Visualize the data spatiotemporally.

**Response to comments**

Thanks for the comments, in the revised versions, we have visualized the spatiotemporal distribution of temperature and BD incidence in descriptive analysis, which are also presented in the following figure (Fig R8):

Fig R8. Descriptive analysis for temperature and BD cases. Panel (a) presents the locations of 31 provinces in China. Panels (b) and (c) present the spatial distributions of average temperatures and total cases of BD in China from 2004 to 2017, respectively. Panel (d) presents the temporal trends of the total BD cases and average temperatures in mainland China. Panel (e) presents the temperature ranges in 31 provinces, where the red horizonal lines indicate the 10%, 30%, 60%, and 90% quantile temperatures across all the province-level monthly temperatures.

4.	Measure and attribute (spatial) stratified heterogeneity, interpret the findings in epidemiology.

**Response to comments**

Thanks for the constructive comments. According to the review’s comments, we have made further analysis about the potential factors which may affect the temperature-BD associations, measured the heterogeneity by using the I^2^ statistics, and explored the spatial difference between different natural zones. The details can be seen in Section 3.2 of the revised version.

Specifically, based on the estimated province-specific associations in the first stage, we incorporated a series of predictors, such as rainfall, relative humidity, poverty, education, GDP and so on, into the multivariate meta-regression to test the potential effect modifiers which may impact the temperature-BD associations in the spatial dimension. The poverty is measured by the proportion of people whose family incomes are below the lowest living standard and the education is measured by the number of pupils per teacher. Due to their significant spatial dependences, the incorporated predictors can control for the spatial confounding in the multivariate meta-regression to some extent. In addition, we also used the multivariate conditional meta autoregression (Wang et al. 2023) to test the residual spatial dependence in the meta-regression and results showed that there is no significant residual spatial dependence, suggesting that the spatial confounding was well controlled.

Seen in Table R2, the I^2^ statistics showed that a considerable heterogeneity exists between provinces. The meta-regressions only with a predictor identified the poverty and rainfall as significant modifier effects (P < 0.05). The P values of relative humidity and education were closed to 0.05. After using an AIC-based forward method to select variables, only poverty and education were included into the multiple meta-regression with P < 0.001, suggesting that poverty and education were two important factors of affecting the temperature-BD associations, which conforms to the expectation since BD is a poverty-related infectious diseases. Furtherly, based on the multiple meta-regression, we plotted the temperature-BD associations at high/low poverty and good/poor education. Shown in Fig R9, the four association curves presented a U-inverted shape; especially at low poverty and good education, the U-inverted shape is obvious. For the associations at high poverty and poor education, the risk of BD rose as temperature increased until temperature was above 29 ℃. In addition, by comparing the slopes in the association curves, the BD incidence at good education seems to be less sensitive to temperature than that at poor education. The reason may be that people with good education have more temperature-related consciousness of protection, thus reducing the impact of temperature on BD incidence.

According to the climate and geography, we also divided the study region into 7 zones and plotted the average association in each zone. Seen in Fig R10, because the subgroup analysis blocks the information borrowing between zones, we obtained the association curve only within the observed temperature range for a specific zone. Overall, the BD incidence rose as temperature increased in all the zones, but when temperature was above 28-30 ℃, apart from the southwest of China, the BD incidence no longer rose and even started to decline especially in the east of China. In the northeast, the association curve presents a flatter slope, suggesting that the BD incidence is less sensitive to temperature; we did not obtain the association at a high temperature (>25℃) due to the observed temperatures less than 25℃.

Table R2. The identification of effect modifiers and heterogeneity tests in multivariate meta-regressions.

| Predictors ^a^ | AIC |  | LR ^b^ |  |  | Q test ^c^ | | | I^2^ |
| --- | --- | --- | --- | --- | --- | --- | --- | --- | --- |
|  |  | Stat | df | *P* |  | Stat | df | *P* |  |
| Intercept | -239.6 | - | - | - |  | 875.1 | 180 | < 0.001 | 79.4 |
| Rainfall | -241.8 | 14.2 | 6 | 0.028 |  | 802.7 | 174 | < 0.001 | 78.3 |
| Humidity | -238.7 | 11.1 | 6 | 0.086 |  | 820.6 | 174 | < 0.001 | 78.8 |
| Sunshine | -237.4 | 9.8 | 6 | 0.135 |  | 813.1 | 174 | < 0.001 | 78.6 |
| GDP | -236.2 | 8.5 | 6 | 0.203 |  | 851.7 | 174 | < 0.001 | 79.6 |
| Wage | -234.1 | 6.4 | 6 | 0.375 |  | 860.1 | 174 | < 0.001 | 79.8 |
| Urbanization | -235.1 | 7.4 | 6 | 0.284 |  | 850.2 | 174 | < 0.001 | 79.5 |
| Poverty | -247.9 | 20.2 | 6 | 0.003 |  | 769.7 | 174 | < 0.001 | 77.4 |
| Education | -239.7 | 12 | 6 | 0.062 |  | 768.4 | 174 | < 0.001 | 77.4 |
| Doctors | -234.1 | 6.4 | 6 | 0.376 |  | 837.6 | 174 | < 0.001 | 79.2 |
| Health worker | -233.4 | 5.7 | 6 | 0.452 |  | 851.9 | 174 | < 0.001 | 79.6 |
| Beds | -235.7 | 8 | 6 | 0.236 |  | 834.3 | 174 | < 0.001 | 79.1 |
| Poverty + education | -250.4 | 34.8 | 12 | < 0.001 |  | 693.1 | 168 | < 0.001 | 75.8 |

Note: ^a^ The detailed explanations for the predictors can be seen in the supplementary materials. Intercept indicates that the multivariate meta-regression includes only an intercept and no predictor. ^b^ The likelihood ratio test was used to test if a predictor significantly modifies the association. ^c^ I^2^ statistic was used to measure the residual heterogeneity and Cochran Q test was used to test the residual heterogeneity.

Fig R9. The temperature-BD associations at different poverty and education. The association curves come from the multivariate meta-regression with poverty and education as predictors. The high and low poverties refer to the proportions of people whose family incomes are below the lowest living standard as 0.135 and 0.007, respectively. The good and poor education refer to the number of pupils per teacher as 11 and 20, respectively.

Fig R10. The associations between temperature and BD incidence in the seven zones.

**Reference**

Wang W, Liao F, Yin F, Ma Y. Mapping the short-term exposure-response relationships between environmental factors and health outcomes and identifying the causes of heterogeneity: A multivariate-conditional-meta-autoregression-based two-stage strategy. Spatial Statistics. 2022:100720.
