# Supplementary material for: Inverted U-shaped association between bacillary dysentery and temperature: A new finding using a novel two-stage strategy in multi-region studies
Source: PLoS Negl Trop Dis. 2023 Nov 17;17(11):e0011771. doi: 10.1371/journal.pntd.0011771 (PMC10691710; doi:10.1371/journal.pntd.0011771)
Supplement: Attachment — Submitted filename: Response to reviewers.docx [file pntd.0011771.s003.docx]

# General reply

Many thanks to the referees for encouraging our work and giving useful comments. We have tried our best to revise the manuscript according to your kind and constructive comments and suggestions. The detailed revisions were marked via the Microsoft word track changes mode and has been uploaded as “Revised manuscript with changes highlighted.docx”. We sincerely hope that this revised manuscript has addressed all your comments and suggestions.

# Point-to-point replies to technical comments

## Editors:

1. An assessment of effect modification was made in Table 1. Please describe the methods and rationale in the Methods section.

**Response to the comments:**

Thanks for the constructive comments. According to the comments, we have given a more detailed description about the methodology of investigating the effect modification, seen in Lines 161-166 and 375-377 of the revised version.

2. Table 1 and R1, “beds” do you mean hospital beds?

**Response to the comments:**

We are sorry for our unclear presentation. We have changed “beds” as “hospital beds“ in Table 1 to enhance the readability.

3. L199, 213, “avoiding the trouble of the singular matrix”. Please use another word for “trouble”

**Response to the comments:**

Thanks for the comments. In the revised version, we have changed “avoiding the trouble of the singular matrix” as “avoiding the singularity of the matrix”, seen in Line 201. Also, we replaced the word “trouble” with “problem” in Line 215 of the revised version.
